# Oxytocin signaling in the ventral tegmental area mediates social isolation-induced craving for social interaction

**DOI:** 10.1186/s12929-025-01130-0

**Published:** 2025-03-17

**Authors:** Hsin-Tzu Chang, Kuan-Hsiang Cheng, Yu-Chieh Hung, Kuei-Sen Hsu

**Affiliations:** 1 Department of Pharmacology, College of Medicine, National Cheng Kung University, No. 1, University Rd., Tainan City, 70101 Taiwan; 2 Institute of Basic Medical Sciences, College of Medicine, National Cheng Kung University, Tainan City, Taiwan

**Keywords:** Oxytocin, Dopamine, Social isolation, Social craving, Ventral tegmental area, Medial prefrontal cortex

## Abstract

**Background:**

Social interaction is crucial for mental health across animal species. Social experiences, especially in early-life stages, strongly influence brain function and social behavior later in life. Acute social isolation (SI) increases motivation to seek social interaction, but little is known about its underlying neuronal and circuitry mechanisms. Here, we focus on oxytocin signaling in the ventral tegmental area (VTA), a vital node of the brain's reward network, as a potential mechanism for SI-induced craving for social interaction.

**Methods:**

Adolescent (4-week-old) or adult (14-week-old) male C57BL/6J mice underwent a 1-week SI. Free interaction, object exploration, three-chamber social approach, and habituation tests were used to assess social and non-social behavior changes. Viral vectors were used to decipher the underlying neural circuitry, and chemogenetic techniques were applied to modify neuronal activity.

**Results:**

We found that in male C57BL/6J mice, SI during adolescence, but not adulthood, leads to increased craving for social interaction and object exploration, accompanied by impaired social habituation, social novelty preference, and social recognition memory (SRM). SI-induced craving for social interaction and SRM deficit is still observed upon regrouping. Through cell-type-specific manipulations with designer receptors exclusively activated by designer drugs (DREADD), we show that oxytocin neurons in the paraventricular nucleus of the hypothalamus (PVN) are crucial for SI-induced social behavior changes. Chemogenetic activation of PVN oxytocin neurons recapitulates social behavior changes observed in SI mice, whereas chemogenetic inhibition of oxytocin neurons prevents social behavior changes caused by SI. Moreover, we found that dopaminergic neurons in the VTA mediate SI-induced craving for social interaction through their projections to the medial prefrontal cortex (mPFC), but not to the nucleus accumbens. Injection of a specific oxytocin receptor antagonist L368,899 into the VTA or chemical lesions of dopaminergic axon terminals in the mPFC with local application of 6-hydroxydopamine ameliorates SI-induced social behavior changes.

**Conclusions:**

These findings suggest that adolescent SI has enduring effects on social behaviors in male mice through an oxytocinergic modulation of the VTA-to-mPFC dopaminergic circuit activity.

**Supplementary Information:**

The online version contains supplementary material available at 10.1186/s12929-025-01130-0.

## Background

Social interaction refers to the ways wherein individuals engage, communicate and respond to one another, and is crucial for establishing and maintaining social behaviors and relationships across various species, including human beings [1–3]. In animals, social interaction can promote an individual’s ability to acquire resources and form alliances that help an individual survive and reproduce [1]. Social interaction is considered a fundamental human need, akin to basic necessities like food and sleep [4–6]. A chronic lack of social interaction was found to be associated with an increased incidence of various neuropsychiatric disorders, such as anxiety, depression, and cognitive impairment [7, 8]. The recent COVID-19 pandemic, caused by the SARS-CoV-2 virus, has significantly reduced social interaction worldwide. Emerging epidemiological evidence suggests that mandated measures, including physical distancing, quarantines, and lockdowns during the COVID-19 pandemic, increased the prevalence of anxiety and depression in adults and adolescents [9–11]. Moreover, people experiencing prolonged social isolation (SI), such as orphans and empty nesters, have reported a higher risk of developing depressive symptoms and insomnia [12, 13]. However, our understanding of the impacts of SI and the mechanisms by which it affects brain structure and function remains incomplete.

Rodent models are undoubtedly helpful for exploring the neurobiological mechanisms behind the effects of SI under highly controlled conditions, including the age and sex of the subject, the duration of isolation, housing conditions, and the developmental time point of isolation [14, 15]. In a brief period of acute SI, such as 24 h, adult mice showed a strong desire to seek social contact and interaction with a novel conspecific [16], whereas, in prolonged periods of chronic SI, such as 2–4 weeks, mice exhibited heightened aggression, avoidance behavior, and social anxiety [17, 18]. Enhanced social motivation to seek social interaction with a novel conspecific is evident in male mice subjected to 1-week SI during adolescence [19], a phenomenon referred to as SI-induced social craving [8]. Similarly, acute SI causes human social cravings analogous to food cravings after fasting [20]. These findings emphasize the rewarding nature of social interaction and suggest that acute SI (periods shorter than 1 week) may be a valuable tool for investigating the rewarding properties of social interaction [21, 22]. While previous work has established dopamine neurons in the dorsal raphe and ventral tegmental area (VTA) as neural substrates responsible for representing the experience of SI [16, 19], we still have limited information about the rewarding properties of social interaction and its underlying neuronal and circuitry mechanisms. The present study seeks to address the existing gap in the literature by focusing on three main questions. First, can SI increase the motivation for social interaction in male mice? Second, does the activity of midbrain VTA dopamine neurons encode the rewarding properties of social interaction? If so, what is the underlying neural circuit mechanism? Finally, as the oxytocin system has been implicated in modulating the salience to social stimuli [23], is oxytocin signaling essential for mediating SI-induced craving for social interaction? Our results revealed that 1-week of adolescent SI leads to increased motivation to seek social interaction in male mice. Mechanistically, oxytocin signaling is a crucial regulator of the VTA-to-medial prefrontal cortex (mPFC) dopaminergic circuit activity that underlies SI-induced craving for social interaction.

## Materials and methods

### Animals

All experiments were conducted in accordance with the National Institutes of Health Guidelines for the Care and Use of Laboratory Animals (NIH publication 865-23) and under protocols approved by the Institutional Animal Care and Use Committee at National Cheng Kung University (Approval No. 110077). Male C57BL/6J mice were used in this study. Oxytocin-Ires-Cre mice (B6;129S-Oxt^tm1.1(cre)Dolsn^/J, Jackson stock #0024234: RRID: IMSR_JAX:024234), in which Cre recombinase is driven by the endogenous oxytocin, were purchased from The Jackson Laboratory and then bred and expanded in our animal facility with C57BL/6J background. Male mice were weaned at postnatal day (P)21 and housed in same-sex littermate groups until P28 in a temperature and humidity-controlled environment (25 ± 1 °C, 50 ± 5%) with a 12 h:12 h light–dark cycle (lights on from 7:00 a.m. to 7:00 p.m.) and ad libitum access to food and water. Mice were randomized into social isolation (SI, one mouse/cage) or grouped housing (GH, three mice/cage) from P28 to P34. To measure the effect of regrouping, mice were housed individually between P28 and P34 and subsequently regrouped until P56. To assess SI in adulthood, mice were housed individually between P98 and P104.

### Stereotaxic viral injections and chemogenetic manipulation

Oxytocin-Ires-Cre mice were anesthetized with 50 mg/kg zolazepam (Zoletil, Virbac) and 5 mg/kg xylazine hydrochloride (Rompun, Bayer) and placed into a stereotactic frame (David Kopf Instruments). Bilateral craniotomies were performed using a 0.5 mm diameter drill, and the virus was injected using a 1 μl Hamilton syringe with a 34-gauge blunt tip needle. A microsyringe pump and its controller were used to maintain the speed of the injection. For chemogenetic activation or silencing of the paraventricular nucleus of the hypothalamus (PVN) oxytocin neurons, GH Oxytocin-Ires-Cre mice were bilaterally injected with AAV_DJ_- hSyn-DIO-hM3D(Gq)-mCherry (0.5 μl at 0.1 μl/min) into the PVN for activation [−0.6 mm anteroposterior (AP), ±0.1 mm mediolateral (ML), −5.0 mm dorsoventral (DV) from Bregma] or SI Oxytocin-Ires-Cre mice were injected bilaterally with AAV_DJ_-hSyn-DIO-hM4D(Gi)-mCherry into the PVN for inhibition. One week after viral injections, SI or GH mice were treated for 1 week with vehicle (4% sucrose and 0.2% saccharin solution) or clozapine-*N*-oxide (CNO, Sigma-Aldrich, Cat#C0832). CNO was dissolved in 200 ml of drinking water at a concentration of 5 mg, in a solution containing 4% sucrose and 0.2% saccharin. The dosage regimen for CNO was chosen based on findings from a previous study [19].

### Cannula implantation and local drug infusion

Cannula implantation and local drug infusion were carried out as described previously [24]. Briefly, mice were anesthetized with zolazepam (50 mg/kg) and xylazine hydrochloride (5 mg/kg) in the stereotaxic frame (David Kopf Instruments) for the entire surgery, and body temperature was maintained with the electrically heating pad. Mice were bilaterally implanted with 26-gauge cannula guides (RWD Life Science Co., Ltd.) aimed at the VTA (−3.0 mm AP, ± 0.6 ML, −4.6 mm DV from Bregma). Cannula guides were kept in place using BioGlue® (CryoLife). Dummy cannulas (30 gauge) were inserted into the guide cannulas to prevent clogging. Mice were intraperitoneally administrated with ketoprofen (5 mg/kg) for postoperative analgesia and given two weeks to recover. L-368,899 (1.25 mM in saline, Tocris Bioscience, Cat#2641) or an equal volume of saline was microinfused bilaterally into the VTA at the rate of 0.25 μl/min (0.5 μl/side) 20 min before behavioral tests [25]. Histological verification of the cannula tip locations was performed at the end of behavioral tests. Only the mice with correctly implanted cannulas (95%) were included in the data analysis.

### In vivo fiber photometry and data analysis

A commercial fiber photometry system (RWD Life Science) was used to record fluorescence signals emitted by GCaMP6s in dopamine neurons in vivo in behaving animals through an optical fiber (diameter, 400 μm, NA, 0.5) implanted in the VTA. AAV_9_-tyrosine hydroxylase (TH)-Cre and AAV_5_-hSyn-DIO-GCaMP6s were infused bilaterally into the VTA of mice. One week following viral injection, mice were subjected to 1-week SI or GH and then underwent a series of behavioral tests. The signal was collected and digitalized at 30 Hz. Change in fluorescence (ΔF/F) was calculated as (F − F0)/F0, where F0 stands for the baseline fluorescence signal. The neuronal activity was also estimated using the area under the curve (AUC) of the Ca^2+^ signal, measured as an integral of the ΔF/F trace over time. The Ca^2+^ signals were aligned with video-scored behavioral events.

### Behavioral tests

All behavioral tests were conducted under dimmed illumination (~10 lx) during the light phase of the day. Mice were acclimated to the testing room for at least 1 h before testing. Different behavioral tasks were conducted in the following sequence: free interaction test, object exploration test, three-chamber social approach test, and habituation test. The direct free interaction task was used to assess social and non-social interactions [19]. The three-chamber social approach task was used for quantitatively measuring sociability, social novelty preference, and social recognition memory (SRM). A short-term habituation task was conducted to assess cognitive ability during repeated stimulation at short intervals. All behavioral tests were performed on separate days, allowing for a 1-day resting interval to prevent potential stress from previous tests. Conducting tests on animal social behaviors every other day does not significantly impact their performance [26, 27]. All behavioral experiments were videotaped for later offline analysis by an experimenter blind to the experimental conditions.

### Direct free interaction task

The subject mouse was placed in a clean home cage (37.5 cm × 17 cm × 18 cm) with fresh bedding and allowed to explore for 10 min freely. After the habituation period, an unfamiliar, sex-matched juvenile conspecific C57BL/6J mouse (3–4 weeks old) or a novel object was placed into the cage for free social interaction for 5 min. Behavior was videotaped, tracked, and analyzed with the EthoVision XT video tracking systems (Noldus; RRID: SCR_000441; Noldus, Wageningen, The Netherlands). This experimental design was implemented to reduce the number of mice used, allowing us to simultaneously gather two interaction score values for the grouped mice [19]. The amount of time spent by the subject mouse in sniffing the stimulus mouse or object was counted as interaction.

### Three-chamber social approach task

The 3-chamber social approach test was conducted as previously described [26]. The social approach apparatus was a polycarbonate rectangle (60 cm × 40 cm × 22 cm) that was divided into three chambers using dividing walls with manual retractable doorways (10 cm × 5 cm) to allow for movement between chambers. The assay consisted of three trials in a quiet and dimly lit (~10 lx) room. The subject mouse was initially placed in the middle chamber and allowed to freely explore all three chambers for 10 min. After habituation, a juvenile male mouse (stimulus, 3–4 weeks old), which had no previous contact with the subject mice, was placed under a wire cage in the left or right chamber (systemically alternated) of the test apparatus and an identical empty wire cage was placed in the opposite side chamber. The subject mouse was placed in the middle chamber and allowed to freely explore all three chambers for 5 min (sociability test). The amount of time spent by the subject mouse in sniffing at the wire cage containing the juvenile stimulus mouse or the empty wire cage was measured. Following the sociability test, the subject mouse was gently guided to the center chamber while a novel, unfamiliar, juvenile male mouse was placed inside the empty wire cage (novel 1). The subject mouse was again allowed to freely explore all three chambers for 5 min to quantify social preference for a novel stranger mouse (social novelty preference test). The subject mouse had a choice between the first, already-investigated mouse (familiar) and the novel unfamiliar mouse. The amount of time spent by the subject mouse in sniffing at the wire cage containing a familiar mouse or a novel mouse was measured. Immediately after the social novelty preference test, the subject mouse was returned to the home cage. The next day, SRM was examined, and the familiar mouse was placed in either the left or right chamber, and a novel unfamiliar juvenile male mouse (novel 2) was placed in the cage of the other side chamber. The subject mouse was free to choose between the already-investigated mouse (familiar) and the novel unfamiliar mouse for 5 min. The amount of time spent by the subject mouse in sniffing at the wire cage containing a familiar mouse or a novel mouse was measured. The behavior of the animals was videotaped and analyzed using the EthoVision XT video tracking system. The discrimination index for assessing the performance of sociability was calculated as [(time of sniffing the stimulus object − time of sniffing the empty cage)/(time of sniffing in both stimulus object and empty cage)]. The discrimination index for assessing the performance of social novelty preference or SRM was calculated as [(time of sniffing the novel object − time of sniffing the familiar object)/(time of sniffing the objects in both novel and familiar object)]. The apparatus was cleaned with 70% ethanol between the testing of each mouse.

### Short-term habituation task

The short-term habituation test was performed as previously described [19]. The subject mouse was initially allowed to freely explore in a clean standard home cage (37.5 cm × 17 cm × 18 cm) with fresh bedding for 10 min. After the habituation period, an unfamiliar conspecific juvenile male mouse (3–4 weeks old) was introduced into the cage for free social interaction for 5 min. At the end of the trial, the stimulus mouse was returned to the home cage, while the subject mouse stayed in the testing cage alone for 10 min. After the inter-trial interval, the same stimulus mouse was returned to the testing cage for another 5 min. The test consisted of four trials with inter-trial intervals of 10 min. Behavior was videotaped, tracked, and analyzed using the EthoVision XT video tracking systems. The amount of time spent by the subject mouse in sniffing the stimulus mouse was measured.

### 6-Hydroxydopamine (6-OHDA) lesion and immunohistochemical assessment

6-OHDA-induced ablation of dopaminergic axon terminals in the mPFC or nucleus accumbens (NAc) was performed as previously described [28]. To prevent dopamine uptake in noradrenaline neurons and increase dopamine uptake by dopamine neurons, mice were pretreated with desipramine (28.5 mg/kg; Sigma-Aldrich, Cat#D3900) and pargyline (6.2 mg/kg; Sigma-Aldrich, Cat#P8013) 30 min before 6-OHDA infusion as previously described [28]. After this injection, mice were anesthetized with a mixture of zolazepam (50 mg/kg) and xylazine hydrochloride (5 mg/kg) in the stereotaxic frame for bilateral intra-mPFC (+1.9 mm AP, ± 0.3 ML, −2.3 mm DV from Bregma) or intra-NAc (+1.4 mm AP, ± 0.85 ML, −4.5 mm DV from Bregma) injections of 6-OHDA (2.5 μg/0.25 μl in 0.2% ascorbic acid in saline, Sigma-Aldrich, Cat#162957). Ascorbic acid was used to prevent the degradation of 6-OHDA. One week after inducing the 6-OHDA lesion, mice were subjected to 1-week SI or GH and subsequently underwent behavioral tests.

On completion of the behavioral testing, mice were anesthetized with 50 mg/kg zolazepam and 5 mg/kg xylazine hydrochloride and then transcardially perfused with 4 °C phosphate buffer solution (PBS) and then 4% paraformaldehyde (PFA) in 0.1 M PBS, pH 7.4, for fixation. Brains were extracted, post-fixed in 4% PFA for 24 h at 4 °C, and then transferred to the solution containing 30% sucrose immersed in 4 °C for 48 h. Coronal slices were sectioned to a 40 μm thickness, washed with 0.4% Triton X-100, and then incubated for blocking with a background-reducing agent (Agilent DAKO, Cat#S3022) for 1 h. After blocking, the sections were incubated with mouse anti-tyrosine hydroxylase (TH; 1:500, Immunostar, Cat# 22941) for 16 h at 4 °C in blocking solution to verify dopamine axon terminals in the mPFC and NAc, dopamine cell bodies in the VTA and other neurons expressing TH throughout the brain. Finally, sections were washed with PBS-T (0.4% Triton X-100 in PBS) and then incubated with secondary Alexa-Fluor conjugated 488 (1:500, Invitrogen, Cat#A11008; RRID: AB_143165) for 1 h at room temperature. Afterward, the sections were collected on separate gelatin-subbed glass slides, rinsed extensively in PBS, and mounted with ProLong Gold Antifade Reagent (Invitrogen). Immunolabelled sections were imaged using an Olympus FluoView FV3000 confocal laser scanning microscope (Tokyo, Japan). Only mice with at least an 80% reduction in TH-immunoreactive axon terminals in the mPFC or NAc were included in the analyses.

For quantification of c-Fos immunopositivity, the sections were incubated with primary antibody against c-Fos (1:500, Cell Signaling Technology, Danvers, MA; Cat#2250; RRID: AB_2247211) or oxytocin (1:2000, Millipore, Billerica, MA; Cat#MAB5296; RRID: AB_11212999). Sections were washed with PBS-T and then incubated with secondary Alexa-Fluor conjugated 488 (1:500, Invitrogen, Cat#A11008; RRID: AB_143165) or Alexa-Fluor conjugated 594 (1:2000, Abcam, Cat#ab150176; RRID: AB_2716250) 1 h at room temperature. The c-Fos^+^ neurons were determined only when cells were colocalized with DAPI or oxytocin staining, and visual-based semiquantitative estimation was applied to every six coronal sections containing the PVN.

### Experimental designs

We did not observe animal deaths during the experiments due to experimental manipulations. In experiment 1, to examine the impact of SI on social interaction during adolescence, a series of behavioral tests were performed to assess the consequences of adolescent SI on different aspects of social behavior in male mice. We also used c-Fos immunoreactivity analysis to determine the role of PVN oxytocin neuron activation in the expression of heightened social interaction in adolescent SI mice. In experiment 2, to investigate whether SI during adolescence has long-lasting impacts on various aspects of social behavior, the SI mice were regrouped and then subjected to behavioral tests. In experiment 3, to verify whether PVN oxytocin projections to VTA dopamine neurons may mediate SI-induced increase in craving for social interaction, we targeted PVN oxytocin neurons for bidirectional chemogenetic modulations. Then, mice were subjected to behavioral tests after SI or GH manipulations. In experiment 4, to determine whether VTA dopamine neuron activity drives an SI-induced increase in craving for social interaction, we used fiber photometry to monitor the calcium transients of VTA dopamine neurons during the social interaction test. We also targeted PVN-to-VTA oxytocin projection neurons for chemogenetic silencing during the SI period. Bilateral intra-VTA injections of vehicle or oxytocin receptor antagonist, L-368,899, were used to confirm the role of oxytocin signaling in mediating the effect of SI via the VTA. In experiment 5, to examine whether dopamine inputs from the VTA to the mPFC or NAc mediate the expression of heightened social interaction in SI mice, we performed chemical lesions of dopaminergic axon terminals specifically in the mPFC or NAc with local application of 6-OHDA. We also performed pathway-specific chemogenetic inhibition of VTA dopamine projection neurons during behavioral tests to explore their role in the expression of heightened social interaction in SI mice. In this series of experiments, mice received intraperitoneal injection of CNO (3 mg/kg) 30 min before the behavioral tests.

### Statistical analysis

Sample sizes were determined based on previous work of a similar nature in our laboratory [29] and calculated using G*Power software with a two-tailed analysis, a significance level set at 0.05, and 80% power, ensuring sufficient power to detect the expected effects while minimizing animal use. No specific randomization method was used. Data are presented as the means ± SEM. Statistics were performed using GraphPad Prism 6 software (GraphPad Software Inc.; RRID: SCR_002798). All datasets were tested for normality using the D’Agostino & Pearson normality test. A two-tailed paired Student’s *t*-test was used for within-group comparison. Two-way ANOVA, two-way repeated measures (RM) ANOVA, and Tukey’s post hoc multiple comparison tests were used to assess differences between multiple groups. The number of animals used is indicated by *n*. Differences between groups were considered statistically significant at *P* < 0.05.

## Results

### Adolescent SI induces social behavior changes during adolescence in male mice

**Fig. 1:**
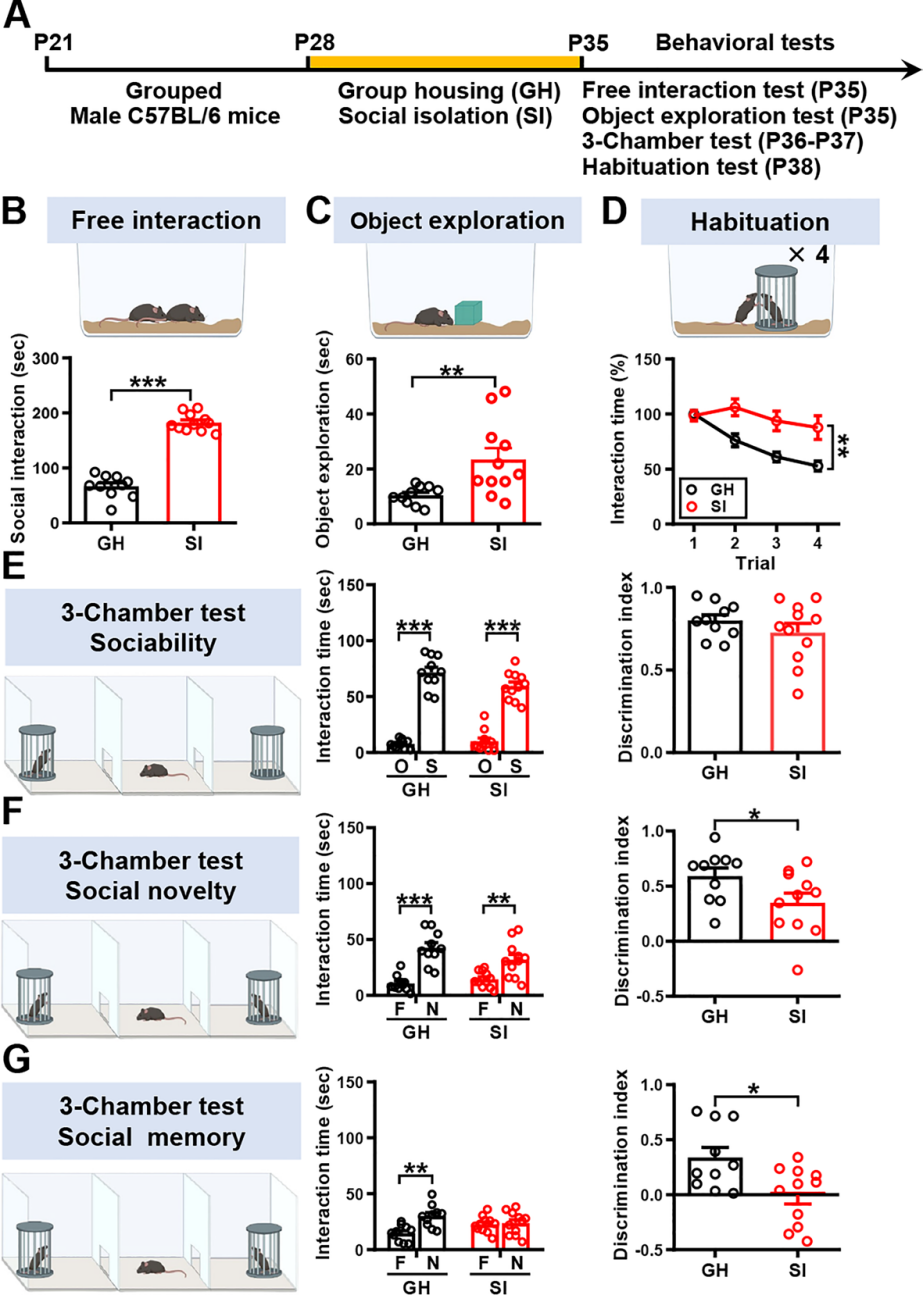
Adolescent SI induces social behavioral changes in male mice. **A** Schematic diagram illustrating the experimental pipeline. After weaning (P21), male mice were housed in groups of the same sex until P27 and housed either in groups or alone between P28 and P34. A series of behavioral tests consisting of the free interaction test, object exploration, 3-chamber test, and habituation test were conducted between P35 and P38. **B** *Top*, schematic representation of the free interaction test. *Bottom*, bar graphs with dots showing the time spent by the male subject mouse in social interaction with a novel mouse. SI mice spend more time interacting with novel mice compared with GH mice [mouse number: GH: *n* = 10; SI: *n* = 11; two-tailed unpaired Student’s *t*-test; *t*_(19)_ = 14.53,* P* < 0.0001, 95% CI (99.32–132.7)]. **C** Top, schematic representation of the object exploration test. Bottom, bar graphs with dots showing the time spent by the subject mouse exploring the novel object. SI mice spend more time exploring the novel object compared with GH mice [mouse number: GH: *n* = 10; SI: *n* = 11; two-tailed unpaired Student’s *t*-test; *t*_(19)_ = 2.94,* P* = 0.0083, 95% CI (3.77–22.31)]. **D** Top, schematic representation of the habituation test. Bottom, scatterplots showing the time spent by the subject mouse in social interaction with the same stimulus mouse for 4 consecutive 5-min trials with an inter-trial interval of 10 min. SI mice exhibited a significant habituation deficit compared with GH mice [mouse number: GH: *n* = 10; SI: *n* = 11; two-way RM ANOVA, trial: *F*_(2.612,49.63)_ = 14.13, *P* < 0.0001; housing condition: *F*_(1,19)_ = 9.47, *P* = 0.0062; trial × housing condition interaction: *F*_(3,57)_ = 6.09, *P* = 0.0011]. **E** *Left*, schematic representation of the 3-chamber sociability test. *Middle*, bar graphs with dots showing the time spent by the male subject mouse in sniffing directed at a wire cage containing the juvenile stimulus mouse (S) or an empty wire cage (E). Both SI and GH subject mice spent significantly more time interacting with the wire cage containing the juvenile stimulus mouse than the empty wire cage. *Right*, the discrimination index (stimulus minus empty) was similar between SI and GH subject mice in the sociability test [mouse number: GH: *n* = 10; SI: *n* = 11; two-tailed unpaired Student’s *t*-test; *t*_(19)_ = 1.10,* P* = 0.28, 95% CI (−0.21 to 0.07)]. **F** *Left*, schematic representation of the 3-chamber social novelty preference test. *Middle*, bar graphs with dots showing the time spent by the male subject mouse in sniffing directed at the wire cage containing a familiar mouse (F) or a novel mouse (N1) 10 min after the sociability test. Both SI and GH subject mice spent significantly more time sniffing the cage containing the novel mouse than the familiar mouse. *Right*, the discrimination index (novel 1 minus familiar) of SI subject mice was significantly less than that of GH subject mice in the social novelty preference test [mouse number: GH: *n* = 10; SI: *n* = 11; two-tailed unpaired Student’s *t*-test; *t*_(19)_ = 2.11,* P* = 0.0485, 95% CI (−0.48 to −0.002)]. **G** *Left*, schematic representation of the 3-chamber SRM test. *Middle*, bar graphs with dots showing the time spent by the male subject mouse in sniffing directed at the wire cage containing a familiar mouse (N1) or a novel 2 mouse (N2), 1 day after the initial interaction. GH, but not SI, subject mice spent significantly more time sniffing the cage containing the novel mouse than the familiar mouse (mouse number: GH: *n* = 10; SI: *n* = 11; paired Student’s* t*-test; *t*_(10)_ = 0.29,* P* = 0.78, 95% CI (−7.30 to 9.52). *Right*, the discrimination index (novel 2 minus familiar) of SI subject mice was significantly less than that of GH subject mice in the SRM test [mouse number: GH: *n* = 10; SI: *n* = 11; two-tailed unpaired Student’s *t*-test; *t*_(19)_ = 2.81,* P* = 0.011, 95% CI (−0.60 to −0.09)]. Data are presented as mean ± SEM. *P < 0.05, **P < 0.01 and ***P < 0.001. Panels **B**–**G** were created with BioRender.com

Since adolescence is a period of heightened affective and social sensitivity [30], we therefore chose to explore the impact of adolescent SI on social interaction. Towards this aim, upon weaning on P21, male mice were group-housed with same-sex littermates until P27 and housed either in groups or alone between P28 and P34. A series of behavioral tests were performed between P35 and P38 to assess the consequences of SI on different aspects of social behavior (Fig. 1A). In the free social interaction test, we found that SI mice spend more time interacting with an unfamiliar conspecific than GH mice (Fig. 1B). In addition, an increase in exploration of the object was observed in SI mice relative to GH mice (Fig. 1C). In the short-term habituation test, GH mice showed significantly decreased interaction time with the same juvenile conspecific for 4 consecutive 2-min exposures each separated by a 10 min inter-trial interval, whereas SI mice showed a significant habituation deficit compared with GH mice (Fig. 1D). In the 3-chamber sociability test, both GH and SI mice exhibited a significant preference for the wire cage containing the stimulus mouse than the empty wire cage, with no difference between housing conditions. The discrimination index was comparable between GH and SI mice in the sociability test (Fig. 1E). In contrast to their normal sociability, SI mice displayed a significant deficit in the social novelty preference test. SI mice had a significantly reduced discrimination index compared with GH mice (Fig. 1F). In the SRM test, we found that SI mice were not able to discriminate between the novel and the familiar mouse as they spent an equal amount of time investigating the novel and the familiar mouse, indicating SI impaired the SRM. GH mice displayed intact SRM, as evidenced by spending significantly more time exploring the novel mouse than the familiar mouse. Consequently, a statistically significant discrimination index was observed between GH and SI in the SRM (Fig. 1G). These results show that adolescent SI increases craving for social interaction and object exploration but impairs social habituation, novelty preference, and SRM in male mice.

### SI during adolescence has a lasting consequence on social interaction

**Fig. 2:**
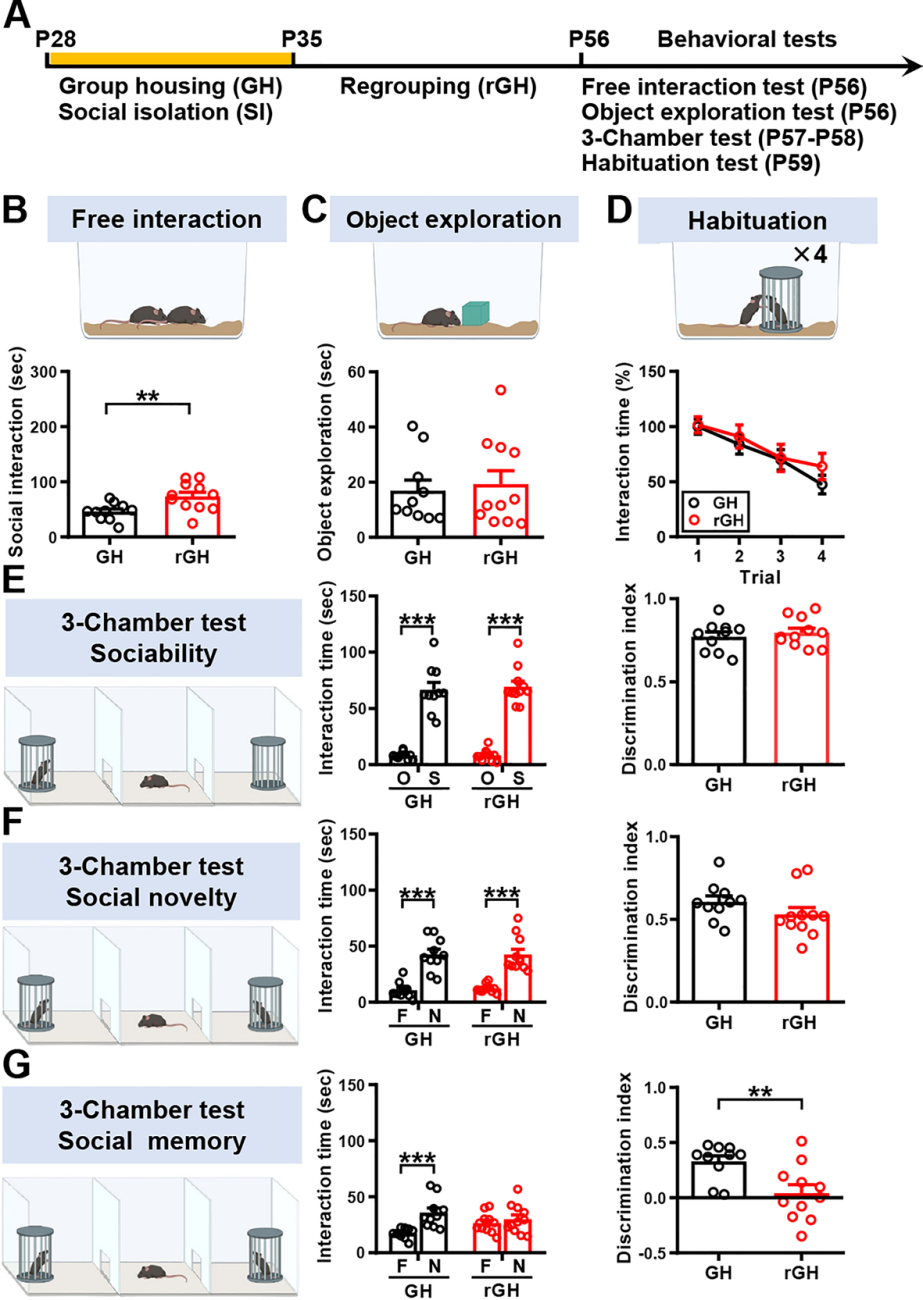
Resocialization is insufficient to rescue receptive social behavioral changes by adolescent SI. **A** Schematic diagram illustrating the experimental pipeline. Male mice were housed either in groups or alone between P28 and P34. SI mice were regrouped (rGH) until P55 and GH mice were always kept in a group. A series of behavioral tests consisting of the free interaction test, object exploration, three-chamber test, and habituation test were performed between P56 and P59. **B** *Top*, schematic representation of the free interaction test. Male rGH mice spend more time interacting with the novel mice compared with male GH mice [mouse number: GH: *n* = 10; SI: *n* = 11; two-tailed unpaired Student’s *t*-test; *t*_(19)_ = 2.91,* P* = 0.0089, 95% CI (7.70–46.98)]. **C** *Top*, schematic representation of the object exploration test. *Bottom*, the time spent exploring the novel object was comparable between male rGH and GH mice [mouse number: GH: *n* = 10; SI: *n* = 11; two-tailed unpaired Student’s *t*-test; *t*_(19)_ = 0.38,* P* = 0.71, 95% CI (−10.69 to 15.47)]. **D** *Top*, schematic representation of the habituation test. *Bottom*, male rGH and GH mice exhibited similar behavioral habituation to repeated social stimulation [mouse number: GH: *n* = 10; SI: *n* = 11; two-way RM ANOVA, trial: *F*_(3,57)_ = 16.49, *P* < 0.0001; housing condition: *F*_(1,19)_ = 0.38, *P* = 0.5472; trial × housing condition interaction: *F*_(3,57)_ = 0.52, *P* = 0.6686]. **E** *Left*, schematic representation of the 3-chamber sociability test. *Middle*, male rGH and GH subject mice spent significantly more time interacting with the wire cage containing the juvenile stimulus mouse (S) than the empty wire cage (E). *Right*, the discrimination index (stimulus minus empty) was comparable between male rGH and GH subject mice in the sociability test [mouse number: GH: *n* = 10; SI: *n* = 11; two-tailed unpaired Student’s *t*-test; *t*_(19)_ = 0.63, *P* = 0.536, 95% CI (−0.06 to 0.11)]. **F** *Left*, schematic representation of the 3-chamber social novelty preference test. *Middle*, male rGH and GH subject mice spent significantly more time sniffing the cage containing the novel mouse (N1) than the familiar mouse (F). *Right*, the discrimination index (novel 1 minus familiar) was comparable between male rGH and GH subject mice in the social novelty preference test [mouse number: GH: *n* = 10; SI: *n* = 11; two-tailed unpaired Student’s *t*-test; *t*_(19)_ = 1.37, *P* = 0.188, 95% CI (−0.20 to 0.04)]. **G** *Left*, schematic representation of the 3-chamber SRM test. Middle, male GH, but not male dGH, subject mice spent significantly more time sniffing the cage containing the novel mouse (N2) than the familiar mouse (N1). Right, the discrimination index (novel 2 minus familiar) of male rGH subject mice was significantly less than male GH subject mice in the SRM test [mouse number: GH: *n* = 10; SI: *n* = 11; two-tailed unpaired Student’s *t*-test; *t*_(19)_ = 3.09,* P* = 0.006, 95% CI (−0.48 to −0.09)]. Data are presented as mean ± SEM. **P < 0.01 and ***P < 0.001. Panels **B**–**G** were created with BioRender.com

To explore whether adolescent SI has long-lasting consequences on different aspects of social behavior, the SI mice were regrouped on P35, and all behavioral tests were performed between P56 and P59 (Fig. 2A). In the free social interaction test, we still observed an increase in social interaction in regrouping SI mice compared with GH mice (Fig. 2B) but object exploration was not affected (Fig. 2C). However, we observed no significant differences in short-term habituation tests between SI and GH mice (Fig. 2D). In comparison with GH mice, we found no effect of adolescent SI in sociability (Fig. 2E) or social novelty preference (Fig. 2F) at P57 following regrouping. However, we still observed a significant deficit in the SRM in regrouping SI mice relative to GH mice (Fig. 2G). These results indicate that adolescent SI induces a lasting increase in craving for social interaction and an impaired SRM performance even with regrouping treatment.

To further evaluate whether SI during adulthood impacts social behavior, male mice were group housed with same-sex littermates until P98 and then housed either in groups or alone between P98 and P104. All behavioral tests were performed between P105 and P106 to assess the consequences of SI during adulthood on different aspects of social behavior (Fig. S1A). We found no effect of SI on social interaction in the free social interaction test (Fig. S1B) or object exploration (Fig. S1C). In addition, we observed no effect of adult SI in behavioral habituation to repeated stimulation in short-term habituation tests (Fig. S1D).

### PVN oxytocin projections to VTA dopamine neurons mediate adolescent SI-induced craving for social interaction

**Fig. 3:**
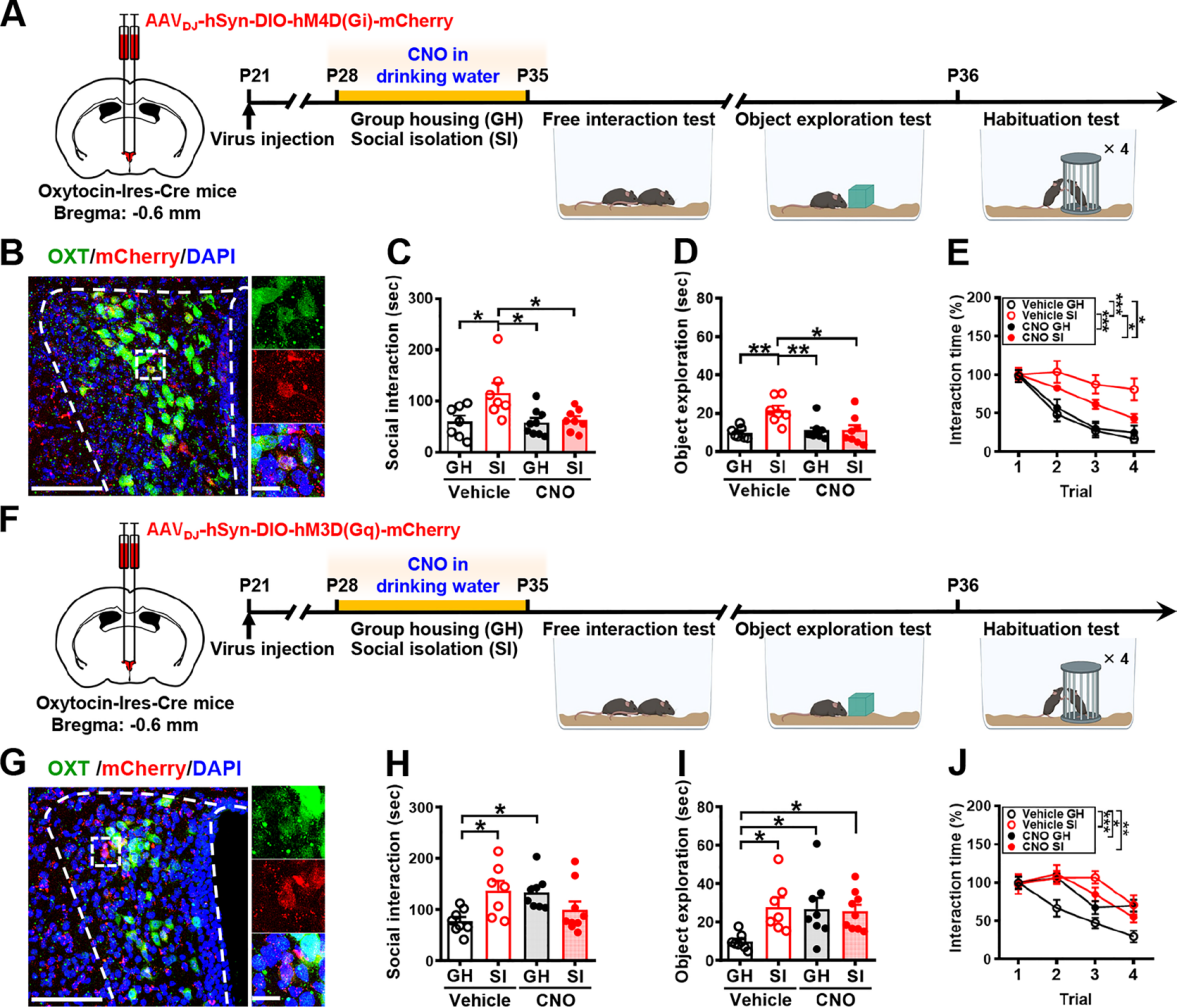
PVN oxytocin projections to VTA dopamine neurons mediate SI-induced craving for social interaction. **A** Schematic representation of the experimental design. AAV_DJ_-hSyn-DIO-hM4D(Gi)-mCherry was bilaterally injected into the PVN of male Oxytocin-Ires-Cre mice. One week after viral infection, mice were housed either in groups or alone, treated with CNO or vehicle in their drinking water for 1 week, and then subjected to the free interaction test, object exploration, and habituation test. **B** Representative images showing the co-expression of hM4D(Gi)-mCherry and oxytocin immunoreactivity in the PVN (*left*). Scale bar, 100 μm. *Right*, magnification of the boxed area; scale bar, 20 μm. **C** Behavioral performance of mice in the free interaction test. There were no significant differences among male vehicle-treated GH, CNO-treated SI, and CNO-treated GH mice in the time spent interacting with a novel mouse [mouse number: GH/vehicle: *n* = 7; SI/vehicle: *n* = 7; GH/CNO: *n* = 9; SI/CNO: *n* = 8; two-way ANOVA, group: *F*_(1,27)_ = 6.183, *P* = 0.0194; treatment: *F*_(1,27)_ = 5.12, *P* = 0.0319; group × treatment interaction: *F*_(1,27)_ = 4.33, *P* = 0.047]. **D** Behavioral performance of mice in the object exploration test. There was a significant difference between male vehicle-treated SI and CNO-treated SI mice while exploring the novel object [mouse number: GH/vehicle: *n* = 7; SI/vehicle: *n* = 7; GH/CNO: *n* = 9; SI/CNO: *n* = 8; two-way ANOVA, group: *F*_(1,27)_ = 7.80, *P* = 0.0095; treatment: *F*_(1,27)_ = 4.72, *P* = 0.0387; group × treatment interaction: *F*_(1,27)_ = 7.45, *P* = 0.011]. **E** Behavioral performance of mice in the habituation test. There was a significant difference between male vehicle-treated SI and CNO-treated SI mice in behavioral habituation to repeated social stimulation [mouse number: GH/vehicle: *n* = 7; SI/vehicle: *n* = 7; GH/CNO: *n* = 9; SI/CNO: *n* = 8; two-way RM ANOVA, trial: *F*_(2.65,71.55)_ = 63.85, *P* < 0.0001; treatment: *F*_(3,27)_ = 8.56, *P* = 0.0004; trial × treatment interaction: *F*_(9,81)_ = 4.39, *P* = 0.0001; Tukey’s post hoc multiple comparisons test, *P*_GH/Vehicle vs. SI/Vehicle_ < 0.0001, *P*_GH/Vehicle vs. SI/CNO_ = 0.0309, *P*_SI/Vehicle vs. GH/CNO_ = 0.0002, *P*_SI/Vehicle vs. SI/CNO_ = 0.0386]. **F** Schematic representation of the experimental design. AAV_DJ_-hSyn-DIO-hM3D(Gq)-mCherry was bilaterally injected into the PVN of male Oxytocin-Ires-Cre mice. One week after viral infection, mice were housed in groups, treated with vehicle or CNO in their drinking water for 1 week, and then subjected to the free interaction test, object exploration, and habituation test. **G** Representative images showing the co-expression of hM3D(Gq)-mCherry and oxytocin immunoreactivity in the PVN (*left*). Scale bar, 100 μm. *Right*, magnification of the boxed area; scale bar, 20 μm. **H** Behavioral performance of mice in the free interaction test. CNO-treated GH mice spend more time interacting with the novel mice compared with vehicle-treated GH mice [mouse number: GH/vehicle: *n* = 8; SI/vehicle: *n* = 7; GH/CNO: *n* = 8; SI/CNO: *n* = 9; two-way ANOVA, group: *F*_(1,28)_ = 0.91, *P* = 0.3475; treatment: *F*_(1,28)_ = 0.53, *P* = 0.4734; group × treatment interaction: *F*_(1,28)_ = 10.92, *P* = 0.0026]. **I** Behavioral performance of mice in the object exploration test. CNO-treated GH mice spend more time exploring the novel object compared with vehicle-treated GH mice [mouse number: GH/vehicle: *n* = 8; SI/vehicle: *n* = 7; GH/CNO: *n* = 8; SI/CNO: *n* = 9; two-way ANOVA, group: *F*_(1,28)_ = 4.22, *P* = 0.0494; treatment: *F*_(1,28)_ = 3.25, *P* = 0.0823; group × treatment interaction: *F*_(1,28)_ = 5.36, *P* = 0.0281]. **J** Behavioral performance of mice in the habituation test. CNO-treated GH mice exhibited a significant habituation deficit compared with vehicle-treated GH mice [mouse number: GH/vehicle: *n* = 8; SI/vehicle: *n* = 7; GH/CNO: *n* = 8; SI/CNO: *n* = 9; two-way RM ANOVA, trial: *F*_(2.746,79.62)_ = 32.64, *P* < 0.0001; treatment: *F*_(3,29)_ = 4.87, *P* = 0.0073; trial × treatment interaction: *F*_(9,87)_ = 3.45, *P* = 0.0011; Tukey’s post hoc multiple comparisons test, *P*_GH/Vehicle vs. SI/Vehicle_ = 0.0007, *P*_GH/Vehicle vs. GH/CNO_ = 0.0107, *P*_GH/Vehicle vs. SI/CNO_ = 0.0099]. Data are presented as mean ± SEM. *P < 0.05, **P < 0.01 and ***P < 0.001. Panels **A** and **F** were created with BioRender.com

Given the proposed importance of oxytocin in governing social interaction, in part through enhancing the salience of social stimuli [31, 32], we next used c-Fos immunoreactivity analysis and chemogenetic manipulations to assess whether increased activation of PVN oxytocin neurons is responsible for the expression of heightened social interaction in male mice subjected to adolescent SI (Fig. S2A). Consistently, we found a significant increase in the percentage of c-Fos^+^oxytocin^+^ neurons in the PVN of SI mice compared to GH mice after the free social interaction session (Fig. S2B, C), whereas no significant difference was found between groups in the percentage of oxytocin^+^ neurons to total DAPI^+^ cells (Fig. S2D). With the goal of chemogenetic manipulations of oxytocin neurons in the PVN, we bilaterally injected AAV_DJ_-hSyn-DIO-hM4D(Gi)-mCherry or control AAV_DJ_-hSyn-DIO-mCherry into the PVN of Oxytocin-Ires-Cre mice [33], in which Cre expression in the PVN is restricted to oxytocin neurons. One week after viral infection, mice were housed in groups or alone and treated with vehicle or CNO in their drinking water for 1 week (P28–P34). Since we asked whether the persistent PVN oxytocin neuron activity is necessary for developing the craving for social interaction and CNO has a short half-life in mice (~1 h) [34], vehicle or CNO was administered in drinking water throughout the GH or SI period to silence neuronal activity. We then used the free social interaction test to examine the social interaction performance (Fig. 3A). Post hoc histological examination of brain sections revealed robust and bilateral co-expression of mCherry with oxytocin in the PVN (Fig. 3B). We confirmed that mCherry was expressed in 65.8 ± 5.6% of PVN oxytocin neurons at 89.7 ± 2.2% accuracy. A two-way ANOVA revealed significant effects for group, treatment, and group treatment interaction (Fig. 3C). Post hoc analysis showed that CNO-treated SI mice spent less time interacting with an unfamiliar conspecific than vehicle-treated SI mice (*P* = 0.0257). In the object exploration test, a two-way ANOVA revealed significant effects for group, effects for treatment, and group × treatment interaction (Fig. 3D). In the short-term habituation test, CNO-treated SI mice exhibited intact behavioral habituation to repeated stimulation compared with vehicle-treated SI mice (Fig. 3E).

As a complementary approach, to examine whether the chronic activation of PVN oxytocin neurons is sufficient to enhance social interaction, we chemogenetic activation of PVN neurons by bilaterally delivering AAV_DJ_-hSyn-DIO-hM3D(Gq)-mCherry into the PVN of Oxytocin-Ires-Cre mice at P21. One week after viral infection, mice were group housed and treated with vehicle or CNO in their drinking water for 1 week (P28–P34) (Fig. 3F). Given that we investigated whether chronic activation of oxytocin neurons in the PVN can enhance the craving for social interaction and CNO has a short half-life in mice [34], vehicle or CNO was administered in drinking water throughout the GH or SI period to enhance neuronal activity. Post hoc histological examination of brain sections revealed robust and bilateral co-expression of mCherry with oxytocin in the PVN (Fig. 3G). We confirmed that mCherry was expressed in 83.6 ± 4.9% of PVN oxytocin neurons at 91.2 ± 1.8% accuracy. In the free social interaction test, a two-way ANOVA revealed a significant effect for group × treatment interaction but no effects for group and treatment (Fig. 3H). Post hoc analysis showed that CNO-treated GH mice spend more time to interact with an unfamiliar conspecific than vehicle-treated GH mice (*P* = 0.0373). In the object exploration test, a two-way ANOVA revealed significant effects for group and group × treatment interaction but no effect for treatment (Fig. 3I). In the short-term habituation test, CNO-treated GH mice exhibited a habituation deficit compared with vehicle-treated GH mice (Fig. 3J).

**Fig. 4:**
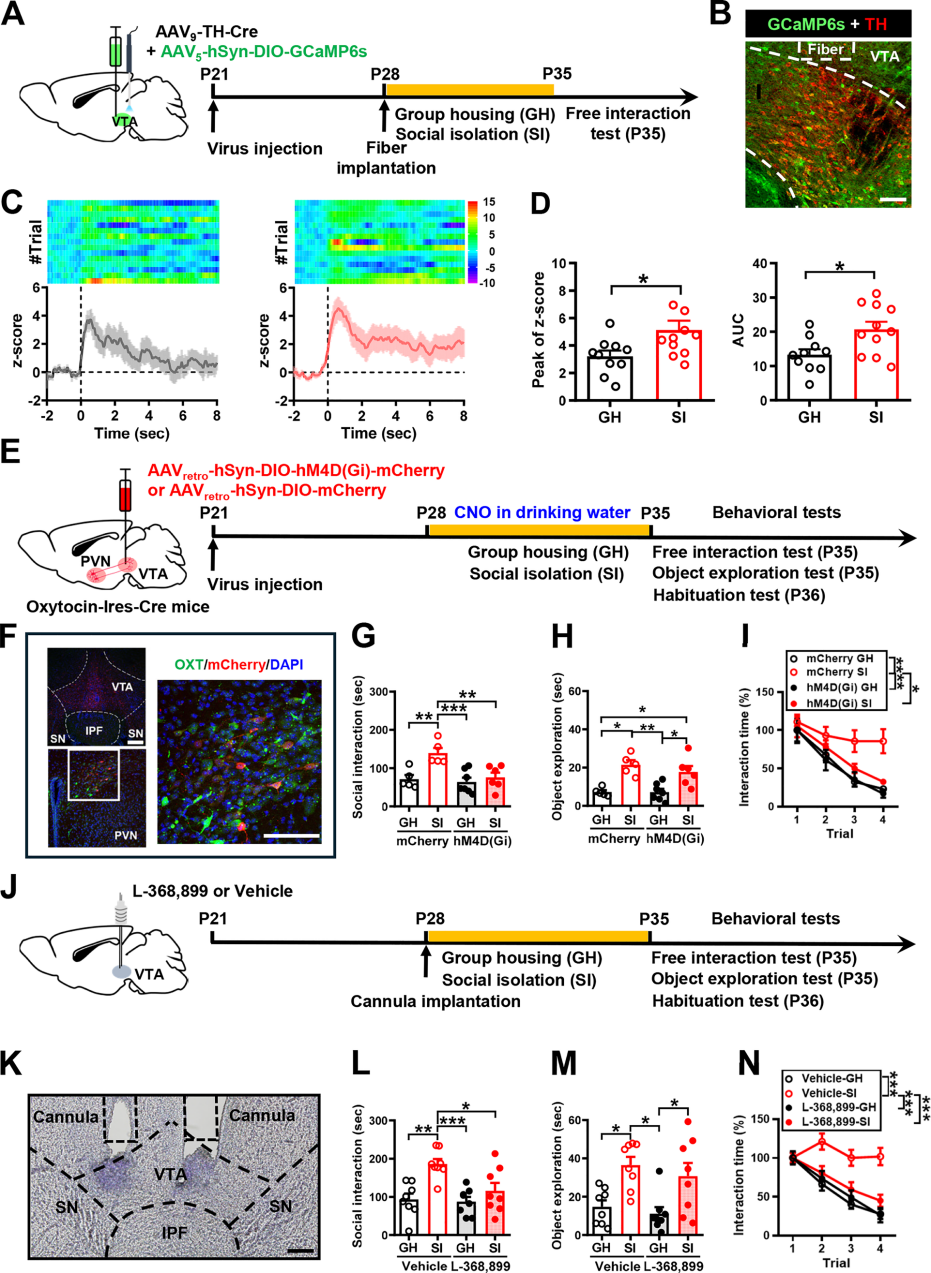
VTA dopamine neuron activity encodes SI-induced craving for social interaction. **A** Schematic representation of the experimental design. AAV_9_-TH-Cre and AAV_5_-hSyn-DIO-GCaMP6s were unilaterally injected into the VTA of male mice and implanted in an optical fiber above the injection site. One week after viral infection, mice were individually housed for 1 week, and then the Ca^2+^ signal of VTA dopamine neurons was recorded at P35 while the subject mouse was engaged in the free social interaction test. **B** Representative image showing Cre-dependent expression of GCaMP6s in VTA dopamine neurons of a mouse with optic fiber placement indicated. Scale bar, 100 μm. **C** Representative heat maps (*top*) and average ΔF/F traces (*bottom*) of dopamine neuron GCaMP6 signals from one example SI mouse and one example of GH mouse aligned to the onset of investigation of a novel conspecific. The trials were obtained from the same GH mouse and SI mouse. **D** Bar graphs with dots showing the quantitative analyses of the peak of Z-score [*left*; mouse number: GH: *n* = 10; SI: *n* = 11; two-tailed unpaired Student’s *t*-test; *t*_(19)_ = 2.33, *P* = 0.03, 95% CI (0.19 to 3.62)] and the area under the curve [UC; *right*; mouse number: GH: *n* = 10; SI: *n* = 11; two-tailed unpaired Student’s *t*-test; *t*_(19)_ = 2.70, *P* = 0.014, 95% CI (1.66 to 13.20)] of the GCaMP signals when male SI or GH mice interacted with the novel mice in the free interaction test. **E** Schematic representation of the experimental design. AAV_retro_-hSyn-DIO-hM4D(Gi)-mCherry or AAV_retro_-hSyn-DIO-mCherry was bilaterally injected into the VTA of male Oxytocin-Ires-Cre mice. One week after viral infection, mice were housed either in groups or alone, treated with CNO in their drinking water for 1 week, and then subjected to the free interaction test, object exploration, and habituation test. **F** Representative images showing the co-expression of hM4D(Gi)-mCherry and oxytocin immunoreactivity in the PVN (*left*). Scale bar, 100 μm. *Right*, magnification of the boxed area; scale bar, 100 μm. **G** Behavioral performance of mice in the free interaction test. There were no significant differences among male mCherry-treated GH, hM4D(Gi)-treated SI, and hM4D(Gi)-treated GH mice in the time spent interacting with a novel mouse [mouse number: GH/mCherry/CNO: *n* = 5; SI/mCherry/CNO: *n* = 5; GH/hM4D(Gi)/CNO: *n* = 7; SI/hM4D(Gi)/CNO: *n* = 6; two-way ANOVA, group: *F*_(1,20)_ = 12.41, *P* = 0.0021; treatment: *F*_(1,20)_ = 9.88, *P* = 0.0051; group × treatment interaction: *F*_(1,20)_ = 6.33, *P* = 0.0205]. **H** Behavioral performance of mice in the object exploration test. There were no significant differences between male mCherry-treated SI and hM4D(Gi)-treated SI mice in the time exploring the novel object [mouse number: GH/mCherry/CNO: *n* = 5; SI/mCherry/CNO: *n* = 5; GH/hM4D(Gi)/CNO: *n* = 7; SI/hM4D(Gi)/CNO: *n* = 6; two-way ANOVA, group: *F*_(1,20)_ = 20.65, *P* = 0.0002; treatment: *F*_(1,20)_ = 0.08, *P* = 0.787; group × treatment interaction: *F*_(1,20)_ = 0.06, *P* = 0.8025]. **I** Behavioral performance of mice in the habituation test. There was a significant difference between male mCherry-treated SI and hM4D(Gi)-treated SI mice in behavioral habituation to repeated social stimulation [mouse number: GH/mCherry/CNO: *n* = 5; SI/mCherry/CNO: *n* = 5; GH/hM4D(Gi)/CNO: *n* = 7; SI/hM4D(Gi)/CNO: *n* = 6; two-way RM ANOVA, trial: *F*_(2.398,45.57)_ = 53.73, *P* < 0.0001; treatment: *F*_(3,19)_ = 4.09, *P* = 0.0213; trial × treatment interaction: *F*_(9,57)_ = 2.20, *P* = 0.0355; Tukey’s post hoc multiple comparisons test, *P*_GH/mCherry/CNO vs. SI/mCherry/CNO_ = 0.005, *P*_SI/mCherry/CNO vs. GH/hM4D(Gi)/CNO_ = 0.0014, *P*_SI/mCherry/CNO vs. SI/hM4D(Gi)/CNO_ = 0.0316]. **J** Schematic representation of the experimental design. Vehicle (saline) or L-368,899 was bilaterally injected into the VTA of male SI or GH mice 20 min before social behavioral tests. **K** Representative coronal section showing bilateral cannula tracks targeting the VTA. Scale bar: 100 μm. **L** Behavioral performance of mice in the free interaction test. There was a significant difference between male vehicle-treated SI and L-368,899-treated SI mice in the time spent interacting with a novel mouse [mouse number: GH/vehicle: *n* = 8; SI/vehicle: *n* = 8; GH/L-368,899: *n* = 8; SI/L-368,899: *n* = 8; two-way ANOVA, group: *F*_(1,28)_ = 14.92, *P* = 0.0006; treatment: *F*_(1,28)_ = 5.86, *P* = 0.0222; group × treatment interaction: *F*_(1,28)_ = 4.25, *P* = 0.0486]. **M** Behavioral performance of mice in the object exploration test. There was no significant difference between male vehicle-treated SI and L-368,899-treated SI mice in the time exploring the novel object [mouse number: GH/vehicle: *n* = 8; SI/vehicle: *n* = 8; GH/L-368,899: *n* = 8; SI/L-368,899: *n* = 8; two-way ANOVA, group: *F*_(1,28)_ = 19.31, *P* = 0.0001; treatment: *F*_(1,28)_ = 1.00, *P* = 0.3242; group × treatment interaction: *F*_(1,28)_ = 0.0536, *P* = 0.8186]. **N** Behavioral performance of mice in the habituation test. There was a significant difference between male vehicle-treated SI and L-368,899-treated SI mice in behavioral habituation to repeated social stimulation [mouse number: GH/vehicle: *n* = 8; SI/vehicle: *n* = 8; GH/L-368,899: *n* = 8; SI/L-368,899: *n* = 8; two-way RM ANOVA, trial: *F*_(2.550,71.41)_ = 48.98, *P* < 0.0001; treatment: *F*_(3,28)_ = 11.08, *P* < 0.0001; trial × treatment interaction: *F*_(9,84)_ = 5.67, *P* < 0.0001; Tukey’s post hoc multiple comparisons test, *P*_GH/Vehicle vs. SI/Vehicle_ < 0.0001, *P*_SI/Vehicle vs. GH/L-368,899_ < 0.0001, *P*_SI/Vehicle vs. SI/L-368,899_ < 0.0001]. Data are presented as mean ± SEM. *P < 0.05, **P < 0.01 and ***P < 0.001

We next identified the brain networks that are functionally activated by PVN oxytocin neurons to promote social interaction following adolescent SI. Given the critical role of VTA dopamine neurons in the processing of social reward [35, 36] and the coding of social interaction [37], we examined whether VTA dopamine neuron activity drives the observed increase in craving for social interactions in adolescent SI mice using fiber photometry calcium imaging coupled with a genetically encoded calcium indicator (GCaMP6s). To obtain a temporal resolution of VTA dopamine neuron activity during social interaction, we used a dual viral approach wherein one virus expresses the Cre recombinase (Cre) gene under the control of the TH promoter (AAV_9_-TH-Cre), thus providing specificity to dopamine neuron and the other expresses a Cre-dependent GCaMP6s (AAV_5_-hSyn-DIO-GCaMP6s) (Fig. 4A). We unilateral injected viral constructs into the VTA of mice and implanted an optical fiber above the injection site (Fig. 4B). One week after viral infection, mice were individually or group housed for 1 week (P28–P34). We then recorded Ca^2+^ signal of VTA dopamine neurons at P35 while the subject mouse was engaged in the free social interaction test. Before recording began, the implanted optic fiber was connected to the photometry system through a jumper cable to simultaneously deliver excitation light and collect real-time tracking of Ca^2+^ signal during the test phase. By aligning the GCaMP signals with video-scored behavioral actions noted in the test phase, we observed behavior-dependent changes in GCaMP signals across social investigation bouts (Fig. 4C). Robust increases in Ca^2+^ signal usually occurred when the subject mouse investigated the novel conspecific. We measured the extent of the responses as the area under the curve and observed a significant difference in the extent of Ca^2+^ signal, with SI mice showing more robust activation of VTA dopamine neurons during investigation compared with GH mice in both the peak of z-score and the area under the curve (Fig. 4D), indicating that, following adolescent SI, the presence of social stimuli is associated with higher VTA dopamine neuron activity.

Following our observations that the activity of PVN oxytocin neurons and VTA dopamine neurons is necessary for the expression of heightened social interaction in adolescent SI mice, we sought to determine whether the oxytocin neurons from the PVN to the VTA dopamine neuron play a role in mediating SI-induced craving for social interaction. To this end, we targeted PVN-to-VTA oxytocin projection neurons for chemogenetic silencing during the SI. Thus, we bilaterally injected AAV_retro_-hSyn-DIO-hM4D(Gi)-mCherry or AAV_retro_-hSyn-DIO-mCherry into the VTA of Oxytocin-Ires-Cre mice. One week after viral infection, mice were individually or group housed and treated with CNO in their drinking water for 1 week (P28–P34) (Fig. 4E). Since we asked whether the persistent activity of PVN-to-VTA oxytocin projection neurons is necessary for developing the craving for social interaction and CNO has a short half-life in mice [34], vehicle or CNO was administered in drinking water throughout the GH or SI period to silence neuronal activity. Post hoc histological examination of brain sections revealed robust and bilateral co-expression of mCherry with oxytocin in the PVN (Fig. 4F). We confirmed that mCherry was expressed in 64.8 ± 5.7% of PVN oxytocin neurons at 94.2 ± 3.8% accuracy. A two-way ANOVA revealed significant effects for group, treatment, and group × treatment interaction (Fig. 4G). Post hoc analysis showed that hM4D(Gi)-treated SI mice spend less time to interact with an unfamiliar conspecific than mCherry-treated SI mice (*P* = 0.0034). In the object exploration test, a two-way ANOVA revealed a significant effect for group but no effects for treatment and group × treatment interaction (Fig. 4H). Post hoc analysis showed no significant difference in object exploration between hM4D(Gi)-treated and mCherry-treated SI mice (*P* = 0.9814). In the short-term habituation test, hM4D(Gi)-treated SI mice exhibited intact behavioral habituation compared with mCherry-treated GH mice. A two-way RM ANOVA revealed significant effects for trial, treatment, and trial × treatment interaction (Fig. 4I).

To further confirm the role of oxytocin signaling in mediating the effect of SI via the VTA, GH, and SI mice were given bilateral intra-VTA injections of vehicle or oxytocin receptor antagonist, L-368,899, 20 min before social behavior tests (Fig. 4J). Post hoc histological analysis revealed bilateral cannula tracks targeting the VTA (Fig. 4K). A two-way ANOVA revealed significant effects for group, treatment, and group × treatment interaction (Fig. 4L). Post hoc analysis showed that L-368,899-treated SI mice spent less time interacting with an unfamiliar conspecific than vehicle-treated SI mice (*P* = 0.0182). In the object exploration test, a two-way ANOVA revealed a significant effect for group but no effects for treatment and group × treatment interaction (Fig. 4M). Post hoc analysis showed no significant difference in object exploration between L-368,899-treated and vehicle-treated SI mice (*P* = 0.8185). In the short-term habituation test, similar to L-368,899-treated GH mice, L-368,899-treated SI mice exhibited intact behavioral habituation to repeated stimulation (Fig. 4N). Altogether, these results demonstrate a major role for PVN oxytocin projections to VTA dopamine neurons in mediating heightened social interaction in adolescent male SI mice.

### Dopamine inputs from the VTA to the mPFC mediate the expression of heightened social interaction in adolescent SI mice

**Fig. 5:**
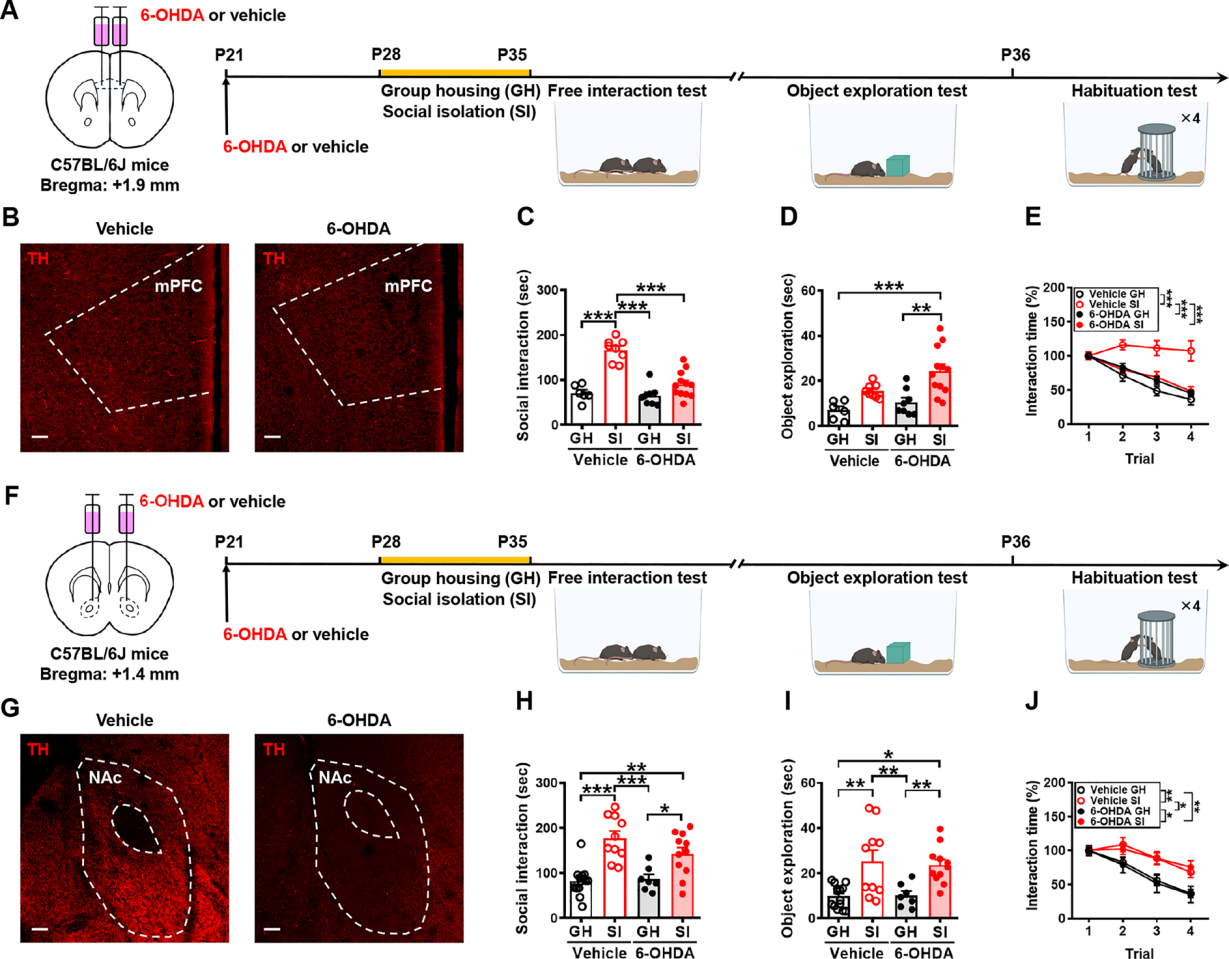
Dopamine release in the mPFC is needed for SI-induced craving for social interaction. **A** Schematic representation of the experimental design. One week after the local injection of vehicle (0.2% ascorbic acid in saline) or 6-hydroxydopamine (6-OHDA) into the mPFC, male mice were housed either in groups or alone for 1 week and then subjected to the free interaction test, object exploration, and habituation test. **B** Representative images of the mPFC illustrating the loss of tyrosine hydroxylase (TH) labeling following ablation of dopaminergic axon terminals by local injection of 6-OHDA compared with vehicle treatment. Scale bars, 100 μm. **C** Behavioral performance of mice in the free interaction test. There were no significant differences among male vehicle-treated GH, 6-OHDA-treated GH and 6-OHDA-treated SI mice in the time spent interacting with a novel mouse [mouse number: GH/vehicle: *n* = 6; SI/vehicle: *n* = 8; GH/6-OHDA: *n* = 8; SI/6-OHDA: *n* = 12; two-way ANOVA, group: *F*_(1,30)_ = 48.15, *P* < 0.0001; treatment: *F*_(1,30)_ = 22.58, *P* < 0.0001; group × treatment interaction: *F*_(1,30)_ = 16.37, *P* = 0.0003]. **D** Behavioral performance of mice in the object exploration test. There was still a significant increase in the time exploring the novel object in 6-OHDA-treated SI mice compared with vehicle-treated GH mice [mouse number: GH/vehicle: *n* = 6; SI/vehicle: *n* = 8; GH/6-OHDA: *n* = 8; SI/6-OHDA: *n* = 12; two-way ANOVA, group: *F*_(1,30)_ = 19.00, *P* = 0.0001; treatment: *F*_(1,30)_ = 5.560, *P* = 0.0251; group × treatment interaction: *F*_(1,30)_ = 1.064, *P* = 0.3106]. **E** Behavioral performance of mice in the habituation test. There was a significant difference between male vehicle-treated SI and 6-OHDA-treated SI mice in behavioral habituation to repeated social stimulation [mouse number: GH/vehicle: *n* = 6; SI/vehicle: *n* = 8; GH/6-OHDA: *n* = 8; SI/6-OHDA: *n* = 12; two-way RM ANOVA, trial: *F*_(2.237,67.10)_ = 36.93, *P* < 0.0001; treatment: *F*_(3,30)_ = 10.49, *P* < 0.0001; trial × treatment interaction: *F*_(9,90)_ = 5.853, *P* < 0.0001; Tukey’s post hoc multiple comparisons test, *P*_GH/vehicle vs. SI/vehicle_ < 0.0001,* P*_SI/vehicle vs. GH/6-OHDA_ < 0.0001, *P*_SI/vehicle vs. SI/6-OHDA_ < 0.0001]. **F** Schematic representation of the experimental design. One week after the local injection of saline or 6-OHDA into the NAc, male mice were housed either in groups or alone for 1 week and then subjected to the free interaction test, object exploration, and habituation test. **G** Representative images of the NAc illustrating loss of TH labeling following ablation of dopaminergic axon terminals by local injection of 6-OHDA compared with vehicle treatment. Scale bars, 100 μm. **H** Behavioral performance of mice in the free interaction test. Male vehicle-treated SI and 6-OHDA-treated SI mice spend more time interacting with the novel mice than male vehicle-treated GH and 6-OHDA-treated GH mice [mouse number: GH/vehicle: *n* = 14; SI/vehicle: *n* = 10; GH/6-OHDA: *n* = 7; SI/6-OHDA: *n* = 11; two-way ANOVA, group: *F*_(1,38)_ = 34.42, *P* < 0.0001; treatment: *F*_(1,38)_ = 1.463, *P* = 0.2339; group × treatment interaction: *F*_(1,38)_ = 2.460, *P* = 0.1251]. **I** Behavioral performance of mice in the object exploration test. Male vehicle-treated SI and 6-OHDA-treated SI mice spend more time exploring the novel object than male vehicle-treated GH and 6-OHDA-treated GH mice [mouse number: GH/vehicle: *n* = 14; SI/vehicle: *n* = 10; GH/6-OHDA: *n* = 7; SI/6-OHDA: *n* = 11; two-way ANOVA, group: *F*_(1,38)_ = 24.44, *P* < 0.0001; treatment: *F*_(1,38)_ = 0.1198, *P* = 0.7312; group × treatment interaction: *F*_(1,38)_ = 0.04, *P* = 0.8341]. **J** Behavioral performance of mice in the habituation test. Male vehicle-treated SI and 6-OHDA-treated SI mice exhibited significant habituation deficits compared with male vehicle-treated GH and 6-OHDA-treated GH mice [mouse number: GH/vehicle: n = 14; SI/vehicle: n = 10; GH/6-OHDA: n = 7; SI/6-OHDA: n = 11; two-way RM ANOVA, trial: *F*_(2.815,107.0)_ = 67.53, *P* < 0.0001; treatment: *F*_(3,38)_ = 4.00, *P* = 0.0143; trial × treatment interaction: *F*_(9,114)_ = 3.83, *P* = 0.0003; Tukey’s post hoc multiple comparisons test, *P*_GH/vehicle vs. SI/vehicle_ = 0.0047, *P*_GH/vehicle vs. SI/6-OHDA_ = 0.0016, *P*_SI/vehicle vs. GH/6-OHDA_ = 0.0284, *P*_GH/6-OHDA vs. SI/6-OHDA_ = 0.0195]. Data are presented as mean ± SEM. *P < 0.05, **P < 0.01 and ***P < 0.001. Panels **A** and **F** were created with BioRender.com

To explore VTA outputs necessary for the expression of heightened social interaction in adolescent SI mice, we focused on the mPFC and NAc that have been identified as downstream targets of the VTA dopamine neurons and potential neuroanatomical substrates involved in motivated non-social and social behaviors [37–39]. To interrogate the role of VTA-to-mPFC pathway in increased craving for social interaction in adolescent SI mice, we performed chemical lesions of dopaminergic axon terminals specifically in the mPFC with local application of vehicle (0.2% ascorbic acid in saline) or 6-OHDA (Fig. 5A). As we investigated whether the release of dopamine in the mPFC is involved in developing the craving for social interaction, vehicle or 6-OHDA was applied 1 week before GH or SI. Post hoc histological examination of brain sections revealed robust loss of TH labeling following 6-OHDA-induced ablation of dopaminergic axon terminals in the mPFC compared with saline treatment (Fig. 5B). One week after inducing the 6-OHDA lesion, mice were singly or group-housed for 1 week and underwent behavioral tests. We observed that ablation of dopaminergic axon terminals in the mPFC significantly prevented SI-induced increases in social interaction in the free social interaction test (Fig. 5C) but not object exploration (Fig. 5D). In the short-term habituation test, similar to saline-treated GH mice, 6-OHDA-treated SI mice exhibited intact behavioral habituation to repeated stimulation (Fig. 5E).

To test whether the VTA-to-NAc pathway is necessary for heightened social interaction in adolescent SI mice, we selectively ablated dopaminergic axon terminals in the NAc with local application of 6-OHDA (Fig. 5F). As we investigated whether the release of dopamine in the NAc is involved in developing the craving for social interaction, vehicle or 6-OHDA was applied 1 week before GH or SI. Post hoc histological examination of brain sections revealed robust loss of TH labeling following 6-OHDA-induced ablation of dopaminergic axon terminals in the NAc. However, ablation of dopaminergic axon terminals in the NAc did not affect SI-induced increases in social interaction in the free social interaction test (Fig. 5H) or object exploration (Fig. 5I). In the short-term habituation test, 6-OHDA-treated SI mice still exhibited a significant habituation deficit to repeated stimulation compared with vehicle-treated GH mice (Fig. 5J).

**Fig. 6:**
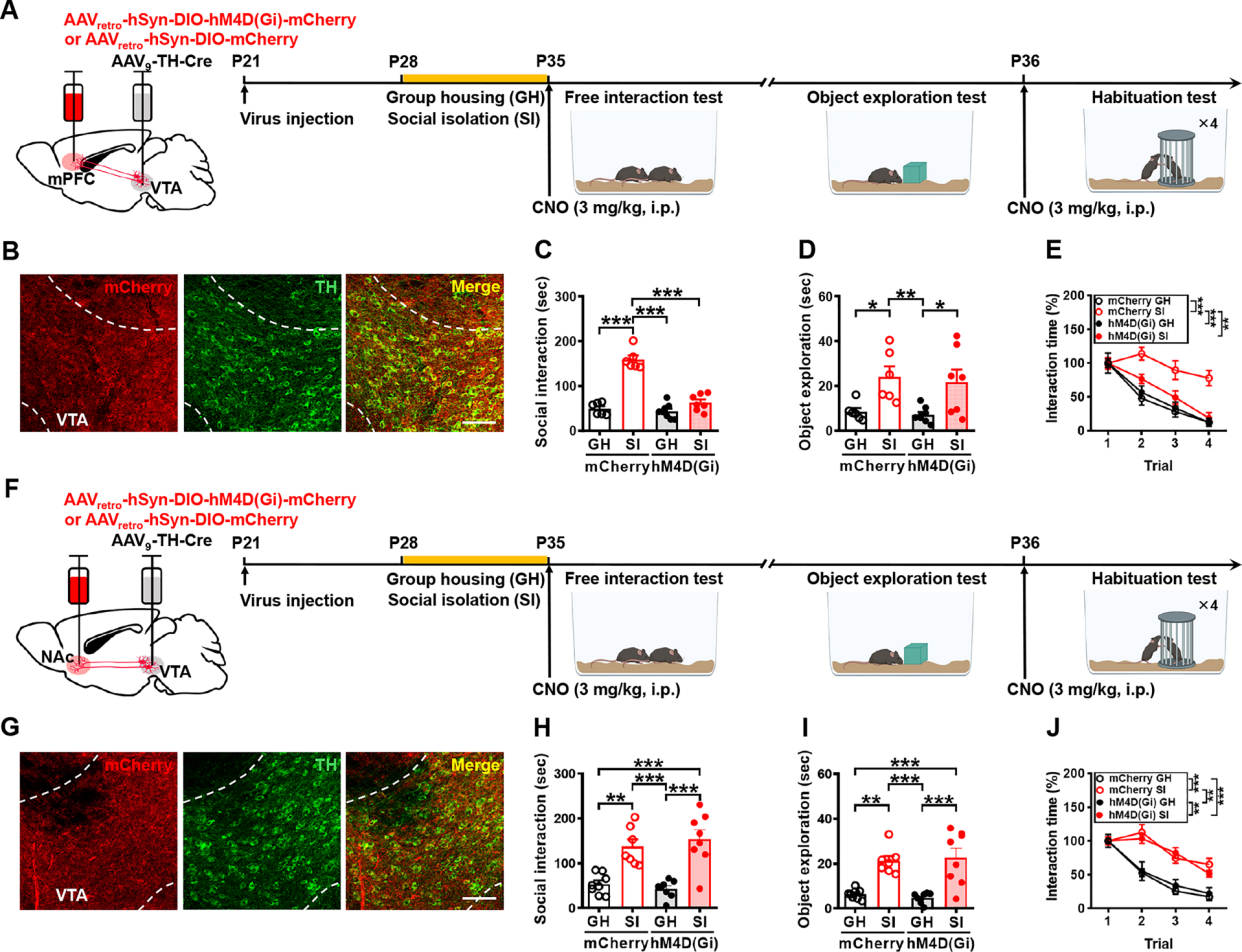
The dopaminergic pathway from the VTA to the mPFC orchestrates the expression of heightened social interaction in SI mice. **A** Schematic representation of the experimental design. AAV_9_-TH-Cre was bilaterally injected into the VTA and AAV_retro_-hSyn-DIO-hM4D(Gi)-mCherry or AAV_retro_-hSyn-DIO-mCherry was bilaterally injected into the mPFC of male mice. One week after viral infection, mice were housed either in groups or alone, and then subjected to the free interaction test, object exploration, and habituation test. CNO (3 mg/kg) was administered intraperitoneally 30 min prior to the behavioral test. **B** Representative images showing the co-expression of hM4D(Gi)-mCherry and TH immunoreactivity in the VTA. Scale bar, 100 μm. **C** Behavioral performance of mice in the free interaction test. There were no significant differences among male mCherry-treated GH, hM4D(Gi)-treated SI, and hM4D(Gi)-treated GH mice in the time spent interacting with a novel mouse [mouse number: GH/mCherry/CNO: *n* = 6; SI/mCherry/CNO: *n* = 6; GH/hM4D(Gi)/CNO: *n* = 8; SI/hM4D(Gi)/CNO: *n* = 7; two-way ANOVA, group: *F*_(1,23)_ = 86.52, *P* < 0.0001; treatment: *F*_(1,23)_ = 53.57, *P* < 0.0001; group × treatment interaction: *F*_(1,23)_ = 42.11, *P* < 0.0001]. **D** Behavioral performance of mice in the object exploration test. There was no significant difference between male mCherry-treated SI and hM4D(Gi)-treated SI mice in the time exploring the novel object [mouse number: GH/mCherry/CNO: *n* = 6; SI/mCherry/CNO: *n* = 6; GH/hM4D(Gi)/CNO: *n* = 8; SI/hM4D(Gi)/CNO: *n* = 7; two-way ANOVA, group: *F*_(1,23)_ = 16.35, *P* = 0.0005; treatment: *F*_(1,23)_ = 0.2534, *P* = 0.6195; group × treatment interaction: *F*_(1,23)_ = 0.02, *P* = 0.8967]. **E** Behavioral performance of mice in the habituation test. There was a significant difference between male mCherry-treated SI and hM4D(Gi)-treated SI mice in behavioral habituation to repeated social stimulation [mouse number: GH/mCherry/CNO: *n* = 6; SI/mCherry/CNO: *n* = 5; GH/hM4D(Gi)/CNO: *n* = 8; SI/hM4D(Gi)/CNO: *n* = 7; two-way RM ANOVA, trial: *F*_(2.694,59.28)_ = 60.26, *P* < 0.0001; treatment: *F*_(3,22)_ = 11.61, *P* < 0.0001; trial × treatment interaction: *F*_(9,66)_ = 3.475, *P* = 0.0015; Tukey’s post hoc multiple comparisons test, *P*_GH/mCherry/CNO vs. SI/mCherry/CNO_ = 0.0002, *P*_SI/mCherry/CNO vs. GH/hM4D(Gi)/CNO_ < 0.0001, *P*_SI/mCherry/CNO vs. SI/hM4D(Gi)/CNO_ = 0.0026]. **F** Schematic representation of the experimental design. AAV_9_-TH-Cre was bilaterally injected into the VTA, and AAV_retro_-hSyn-DIO-hM4D(Gi)-mCherry or AAV_retro_-hSyn-DIO-mCherry was bilaterally injected into the NAc of male mice. One week after viral infection, mice were housed either in groups or alone, and then subjected to the free interaction test, object exploration, and habituation test. CNO was administered intraperitoneally 30 min prior to the behavioral test. **G** Representative images showing the co-expression of hM4D(Gi)-mCherry and TH immunoreactivity in the VTA. Scale bar, 100 μm. **H** Behavioral performance of mice in the free interaction test. Male mCherry-treated SI and hM4D(Gi)-treated SI mice spend more time interacting with the novel mice compared with male mCherry-treated GH and hM4D(Gi)-treated GH mice [mouse number: GH/mCherry/CNO: *n* = 8; SI/mCherry/CNO: *n* = 7; GH/hM4D(Gi)/CNO: *n* = 8; SI/hM4D(Gi)/CNO: *n* = 8; two-way ANOVA, group: *F*_(1,27)_ = 47.98, *P* < 0.0001; treatment: *F*_(1,27)_ = 0.05, *P* = 0.8335; group × treatment interaction: *F*_(1,27)_ = 0.95, *P* = 0.3374]. **I** Behavioral performance of mice in the object exploration test. Male mCherry-treated SI and hM4D(Gi)-treated SI mice spend more time exploring the novel object compared with male mCherry-treated GH and hM4D(Gi)-treated GH mice [mouse number: GH/mCherry/CNO: *n* = 8; SI/mCherry/CNO: *n* = 7; GH/hM4D(Gi)/CNO: *n* = 8; SI/hM4D(Gi)/CNO: *n* = 8; two-way ANOVA, group: *F*_(1,27)_ = 44.50, *P* < 0.0001; treatment: *F*_(1,27)_ = 0.0008, *P* = 0.9773; group × treatment interaction: *F*_(1,27)_ = 0.42, *P* = 0.5222]. **J** Behavioral performance of mice in the habituation test. Male mCherry-treated SI and hM4D(Gi)-treated SI mice exhibited significant habituation deficits compared with male mCherry-treated GH and hM4D(Gi)-treated GH mice [mouse number: GH/mCherry/CNO: *n* = 8; SI/mCherry/CNO: *n* = 7; GH/hM4D(Gi)/CNO: *n* = 8; SI/hM4D(Gi)/CNO: *n* = 8; two-way RM ANOVA, trial: *F*_(2.430,65.62)_ = 77.81, *P* < 0.0001; treatment: *F*_(3,27)_ = 12.43, *P* < 0.0001; trial × treatment interaction: *F*_(9,81)_ = 5.631, *P* < 0.0001; Tukey’s post hoc multiple comparisons test, *P*_GH/mCherry/CNO vs. SI/mCherry/CNO_ = 0.0001, *P*_GH/mCherry/CNO vs. SI/hM4D(Gi)/CNO_ = 0.0002, *P*_SI/mCherry/CNO vs. GH/hM4D(Gi)/CNO_ = 0.0017, *P*_GH/hM4D(Gi)/CNO vs. SI/hM4D(Gi)/CNO_ = 0.0036]. Data are presented as mean ± SEM. *P < 0.05, **P < 0.01 and ***P < 0.001. Panels **A** and **F** were created with BioRender.com

To further confirm that the dopaminergic pathway from the VTA to the mPFC orchestrates the expression of heightened social interaction in adolescent SI mice, we used pathway-specific chemogenetic inhibition of VTA dopamine neurons during the behavioral tests. We bilaterally injected AAV_9_-TH-Cre into the VTA and AAV_retro_-hSyn-DIO-hM4D(Gi)-mCherry or control AAV_retro_-hSyn-DIO-mCherry into the mPFC of mice. One week after viral infection, mice were individually housed for 1 week and then subjected to social behavioral tests following intraperitoneal injection of CNO (3 mg/kg) (Fig. 6A). Since we asked whether the activity of the VTA-to-mPFC dopaminergic pathway is involved in orchestrating the expression of heightened social interaction, CNO was administered intraperitoneally 30 min prior to the behavioral test to inhibit neuronal pathway activity. As CNO has a short half-life in mice [34], CNO was given on P35 and P36 before the behavioral tests. We confirmed that mCherry was expressed in 64.8 ± 4.1% of VTA dopamine neurons at 93.6 ± 2.8% accuracy (Fig. 6B). We observed that chemogenetic inhibition of the VTA-to-mPFC dopaminergic pathway significantly prevented SI-induced increases in social interaction in the free social interaction test (Fig. 6C) but not object exploration (Fig. 6D). In the short-term habituation test, similar to mCherry-treated GH mice, hM4D(Gi)-treated SI mice exhibited intact behavioral habituation to repeated stimulation (Fig. 6E). To test the role of the dopaminergic pathway from the VTA to the NAc in mediating heightened social interaction in adolescent SI mice, we bilaterally injected AAV_5_-TH-Cre into the VTA and AAV_retro_-hSyn-DIO-hM4D(Gi)-mCherry or control AAV_retro_-hSyn-DIO-mCherry into the NAc of mice. One week after viral infection, mice were individually housed for 1 week and then subjected to social behavioral tests following intraperitoneal injection of CNO (3 mg/kg) (Fig. 6F). Since we asked whether the activity of the VTA-to-NAc dopaminergic pathway is involved in orchestrating the expression of heightened social interaction, CNO was administered intraperitoneally 30 min prior to the behavioral test. As CNO has a short half-life in mice [34], CNO was given on P35 and P36 before the behavioral tests. We confirmed that mCherry was expressed in 63.4 ± 4.6% of VTA dopamine neurons at 91.4 ± 1.8% accuracy (Fig. 6G). By contrast, chemogenetic inhibition of the VTA-to-NAc dopaminergic pathway had no effects on SI-induced increases in social interaction in the free social interaction test (Fig. 6H) or object exploration (Fig. 6I). In the short-term habituation test, CNO-treated hM4D(Gi)-mCherry-expressed SI mice still exhibited a significant habituation deficit to repeated stimulation (Fig. 6J). Collectively, these results establish a vital role for the VTA-to-mPFC dopaminergic pathway in orchestrating the expression of heightened social interaction in adolescent male SI mice.

## Discussion

Despite the fundamental importance of social experiences during adolescence in brain function and building social behavior later in life [40–42], little is known about how social interaction generates rewarding experiences and the neural circuit mechanisms underlying its rewarding properties. In the present study, we combined chemogenetic manipulations, pharmacological treatment, fiber photometry calcium imaging, and behavioral assays to unravel the lasting consequences of adolescent SI on social behaviors in male C57BL/6J mice. We show that 1-week adolescent SI leads to an increased craving for social interaction and object exploration but impairs behavioral habituation to repeated social stimulation, social novelty preference, and SRM. Our data indicate that PVN oxytocin projections to VTA dopamine neurons are associated with the development of SI-induced craving for social interaction. Importantly, our results affirm that social interaction has rewarding aspects and engages neural circuits of motivation, specifically the VTA dopamine projections to the mPFC. Together, these results support a causal role for oxytocin signaling in the VTA in boosting rewarding properties of social interaction and uncover the importance of the VTA-to-mPFC dopaminergic pathway in orchestrating the expression of heightened social interaction in adolescent male SI mice.

The SI affects brain function and behavior differently based on experience duration [8, 22]. There is substantial evidence that prolonged periods of social isolation have harmful effects on the physiology and behavior of individuals in both humans and animals [15, 18, 43]. However, recent studies in rodents have highlighted the positive prosocial effects of brief periods of acute SI [16, 19, 41, 44]. Parallel to this finding, a recent human study found that an acute 10-h SI evokes midbrain craving responses to social cues, akin to how fasting induces feelings of hunger [6]. Our findings align with previous work showing that 1-week adolescent SI leads to increased motivation to seek social contact with novel conspecifics in male mice [19]. Additionally, we did not observe a significant impact of SI during adulthood on motivation to seek social contact, suggesting that adolescence is particularly important for generating this behavioral rebound effect. The reasons for the varying outcomes of social interaction during adolescence and adulthood are still unknown, but one potential interpretation, based on social homeostasis theory [21, 44], is that the homeostatic set point for social contact may differ between adolescents and adults, potentially making adolescents more vulnerable than adults to express SI-induced rebound effect on social interaction. It is worth noting that while the SI paradigm used in this study is based on the model proposed by Musardo et al. [19], our results do not fully align with their findings. Contrary to the findings of Musardo et al. [19], who showed no effects, our data indicate that SI during adolescence also boosts object exploration and hinders behavioral habituation to repeated stimulation over time. It is unlikely that the observed effects are due to the influence of prior experience in the social interaction test because the same results were found in SI mice without any prior behavioral testing. The reasons for the inconsistent observations are unclear, but they may be related to subtle differences in animal handling or other unrecognized factors. One unexpected finding is that adolescent SI also results in increased object exploration. Although further studies are warranted to dissect the neural basis underlying this enhancement, in contrast to SI-induced craving for social interaction, targeting the VTA-to-mPFC or VTA-to-NAc dopaminergic pathways does not influence the increased object exploration following SI. We speculate that different neural circuit pathways are activated to encode changes in social and non-social behaviors induced by SI. Although the social interaction test and the object exploration test were conducted consecutively without a resting interval, the increased object exploration is not due to the potentially stressful nature of the prior social interaction test. Indeed, the same result was observed in SI mice without a prior social interaction test (data not shown). Moreover, our results confirm and extend previous findings that adolescent SI results in impaired social novelty preference and SRM without changing sociability in the 3-chamber test [19, 41]. This suggests that the SI-induced rebound effect on social interaction may hinder the ability to distinguish familiar and novel conspecifics, resulting in a decreased preference for social novelty and SRM. Our findings also align with the work by Almeida-Santos et al. [45], which found that 1-week SI impairs the persistence of SRM by disturbing glutamatergic tonus and communication between the olfactory bulb and hippocampus. Additionally, impaired preference for social novelty may account for the observed SI-induced SRM deficit.

The neuropeptide oxytocin is essential for refining social cognition and behavior in mammals [31, 46, 47]. Several lines of evidence have shown that oxytocin can enhance the salience of social stimuli, the subsequent approach and manifestations of social behavior, and the rewarding aspects of social interactions in selected reproductive and non-reproductive social behaviors [25, 32, 48, 49]. We demonstrated the necessity of oxytocin signaling for SI-induced increase in social interaction in mice by administrating oxytocin receptor antagonist L-368,899 into the VTA, thereby reducing motivation to seek out social contact with novel conspecifics. To demonstrate the role of the central oxytocin system in mediating SI-induced increases in craving for social interaction, we conditionally induced designer receptors exclusively activated by designer drugs (DREADD) expression in oxytocin neurons in the PVN. We observed that chemogenetic inhibition of PVN oxytocin neurons during SI ameliorated the observed rise in social interaction in SI mice, whereas chemogenetic activation of PVN oxytocin neurons produced an opposite increase in social interaction in GH mice (Fig. 3), strongly implying that PVN oxytocin signaling is necessary and sufficient for SI-induced social craving. Our findings are consistent with previous studies showing that SI can lead to changes in the central oxytocin system, which subsequently affects social behaviors [19, 50, 51]. We further expand on these by highlighting a crucial role in the loss of social contact during adolescence. As there are numerous routes through which oxytocin is released and reaches its target brain regions [52, 53], future studies are needed to determine whether SI-induced increase in oxytocin levels in the VTA results from either axonal release from PVN oxytocin neurons or indirectly from the bloodstream. Furthermore, further investigation is needed to explore how adolescent SI increases PVN oxytocin signaling.

We also explored the neural substrates underlying the effect of oxytocin on social interactions. As recent studies indicate that VTA dopamine neuron activity encodes social interaction [37] and adolescent SI induces hyperactivity of VTA dopamine neurons [19], we examined whether VTA dopamine neurons and their projections to the mPFC or NAc drive SI-induced increase in motivation to seek out social interaction. Our findings that the extent of rise in VTA dopamine neuron activity of SI mice during social interaction was significantly higher than that of GH mice and that chemogenetic silencing of PVN-to-VTA oxytocin projection neurons during SI period or pharmacological blockade of VTA oxytocin receptors prevented the expression of heightened social interactions in adolescent SI mice suggest that PVN oxytocin neurons mediated the social craving effect of SI via the VTA dopamine neurons [19]. Furthermore, although the VTA is known to send dense dopaminergic projections to both the mPFC and NAc to motivate non-social or social behaviors [37–39], our data of 6-OHDA-induced axon terminal lesion and circuit-specific chemogenetic silencing identified the VTA-to-mPFC dopaminergic pathway as the primary reward circuit in orchestrating SI-induced social craving responses. Our findings are in line with earlier literature showing that dopaminergic projections from the VTA to the mPFC and NAc are functionally different in the regulation of depressive-like and nociceptive behaviors [53–55] and further expand on these by implicating a vital role for the VTA-to-mPFC dopaminergic pathway in maintaining social homeostasis [21, 56]. Since we investigated whether the release of dopamine in the mPFC is involved in developing the craving for social interaction, 6-OHDA was administered 1 week prior to either GH or SI. In this study, although we did not examine the effect of 6-OHDA treatment when applied after GH or SI, we speculate that this treatment may reduce the expression of heightened social interaction in SI mice. This interpretation is based on our observation that chemogenetic inhibition of the VTA-to-mPFC dopaminergic pathway effectively reduced SI-induced increases in social interaction in the free social interaction test (Fig. 6C). Additional study is needed to explore this possibility.

A key finding of this study was that SI during adolescence has lasting effects. Our finding that adolescent SI mice spent more time in contact with the conspecific than GH mice did 3 weeks after regrouping aligns with the observation of Musardo et al. [19], who found that SI has acute and long-lasting effects on social interaction accompanied by changes in excitatory synaptic transmission onto VTA dopamine neurons. Moreover, our finding that SI mice showed an SRM deficit, even after 3 weeks of regrouping, is consistent with the finding that juvenile SI showed a social recognition deficit in mice in adulthood after resocialization [57], although the durations of SI are different between the two studies (1-week vs. 8-weeks). Because we found that regrouping can reverse SI-induced changes in object exploration, social habituation, and social novelty preference, it is plausible that some, but not all, consequences of adolescent SI are long-lasting and cannot be reversed through resocialization.

There are three limitations in our study. First, the lack of female mice used in this study limits our findings, as we cannot determine whether the identified neuronal and circuitry mechanisms apply to both sexes. Our preliminary data indicated that female SI mice spent more time interacting with an unfamiliar conspecific compared to female GH mice in the free social interaction test (data not shown). However, we did not investigate whether SI influences social behaviors through similar neuronal and circuitry mechanisms involving the oxytocin signaling pathway in both sexes. As oxytocin levels and receptor expression may differ between sexes and fluctuate during the estrus cycle [58–60], more detailed experimental designs are necessary to determine whether adolescent SI induces changes in social behavior in both sexes through the same or different neuronal and circuitry mechanisms. Second, we do not know how SI imprints on the PVN oxytocin system, and the molecular mechanisms by which oxytocin induces a long-lasting increase in VTA dopamine neuron activity upon SI treatment need to be further explored. It was found that SI-induced increase in social interaction is associated with increased insertion of GluA2-lacking AMPA receptors at excitatory synapses onto VTA dopamine neurons [19]. Further studies are required to uncover the role of oxytocin in mediating this effect. Third, although we focus primarily on SI-induced craving for social interaction in this study, we also found that SI during adolescence increases non-social object exploration in later life. This is a highly intriguing finding that merits further dedicated research.

## Conclusions

This study unravels a causal link between oxytocin-driven enhancement in the activity of VTA dopamine neurons and an increased craving for social interaction in adolescent SI mice. Our results reveal that 1-week of SI during adolescence causes a lasting increase in craving for social interaction in male mice. We further identify a crucial role of the VTA-to-mPFC dopaminergic pathway in orchestrating the expression of this rebound effect. While it remains to be seen whether these findings have translational validity in humans, our current studies provide more insights into oxytocin signaling as a critical regulator of the neural substrates underlying reinforcing properties of social interaction and advance our understanding of the neurobiological mechanisms underlying maintaining social homeostasis.

## Supplementary Information

Additional file 1.

## Data Availability

The authors confirm that all data generated and analyzed during this study are either included in this published article or available from the corresponding authors upon reasonable request.

## Abbreviations

AAV: Adeno-associated virus
ANOVA: Analysis of variance
AP: Anteroposterior
CNO: Clozapine-*N*-oxide
DREADD: Designer receptor exclusively activated by designer drugs
DV: Dorsoventral
GH: Group-housed
ML: Mediolateral
mPFC: Medial prefrontal cortex
6-OHDA: 6-Hydroxydopamine
NAc: Nucleus accumbens
P21: Postnatal day 21
PBS: Phosphate buffer solution
PFA: Paraformaldehyde
PVN: Paraventricular nucleus of the hypothalamus
RM: Repeated measures
SI: Social isolation
SRM: Social recognition memory
TH: Tyrosine hydroxylase
VTA: Ventral tegmental area

## Availability of data and materials

All data generated or analyzed during this study are included in the published article.
