# Supplementary material for: Oxytocin signaling in the ventral tegmental area mediates social isolation-induced craving for social interaction
Source: J Biomed Sci. 2025 Mar 17;32:37. doi: 10.1186/s12929-025-01130-0 (PMC11912778; doi:10.1186/s12929-025-01130-0)
Supplement: Supplementary file 1 — Additional file 1. [file 12929_2025_1130_MOESM1_ESM.pdf]

Supplementary material

Oxytocin signaling in the ventral tegmental area mediates social isolation-induced craving for social interaction

Hsin-Tzu Chang[1,†], Kuan-Hsiang Cheng[2,†], Yu-Chieh Hung[2,†], Kuei-Sen Hsu[1,2,*]

[1] Department of Pharmacology, College of Medicine, National Cheng Kung University, Tainan, Taiwan

[2] Institute of Basic Medical Sciences, College of Medicine, National Cheng Kung University, Tainan, Taiwan

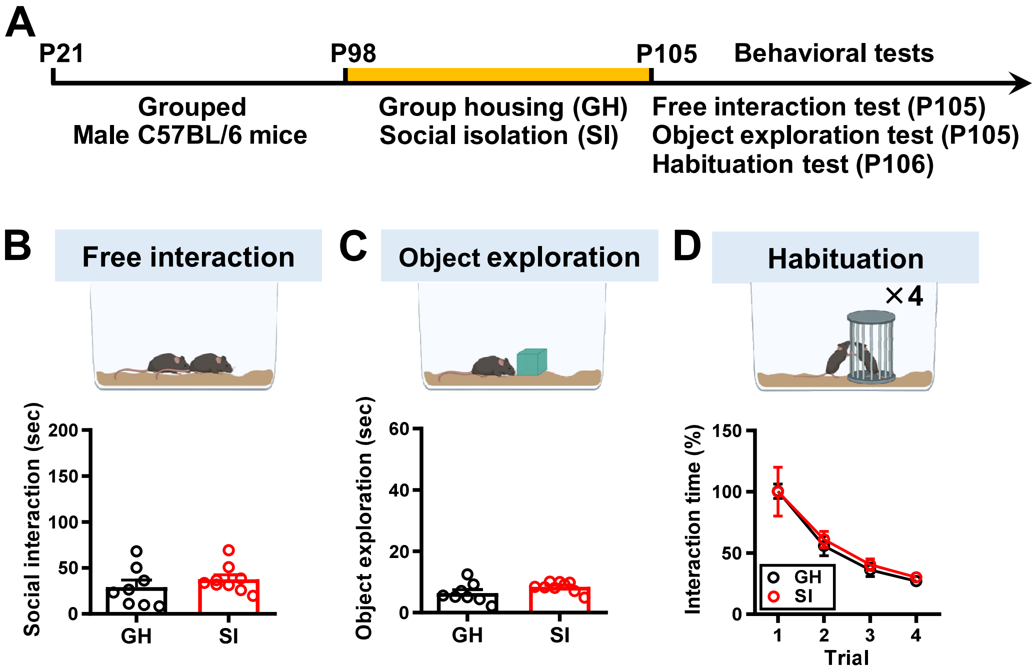

Supplementary Figure S1 SI during adulthood does not influence free social interaction or object exploration. **A** Schematic diagram illustrating the experimental pipeline. After weaning (P21), male mice were housed in groups of the same sex until P83 and then housed either in groups or alone between P98 and P104. A series of behavioral tests consisting of the free interaction test, object exploration and habituation test were performed between P105 and P106. ***B***, Top, schematic representation of the free interaction test. There was no significant difference between male SI and GH mice in the time spent interacting with a novel mouse [mouse number: GH: $n = 8$; SI: $n = 9$; two-tailed unpaired Student's *t*-test; $t_{(15)} = 0.95$, $P = 0.357$, 95% CI (-10.40 to 27.13)]. ***C***, Top, schematic representation of the object exploration test. Bottom, the time spent exploring the novel object was comparable between male SI and GH mice [mouse number: GH: $n = 8$; SI: $n = 9$; two-tailed unpaired Student's *t*-test; $t_{(15)} = 1.67$, $P = 0.115$, 95% CI (-0.56 to 4.62)]. ***D***, Top, schematic representation of the habituation test. There was no significant difference between male SI and GH in the habituation test [mouse number: GH: $n = 8$; SI: $n = 9$; two-way RM ANOVA, trial: $F_{(3,45)} = 35.42$, $P < 0.0001$; housing condition: $F_{(1,15)} = 0.11$, $P = 0.7399$; trial × housing condition interaction: $F_{(3,45)} = 0.05$, $P = 0.9836$]. Panels B, C, and D were created with BioRender.com.

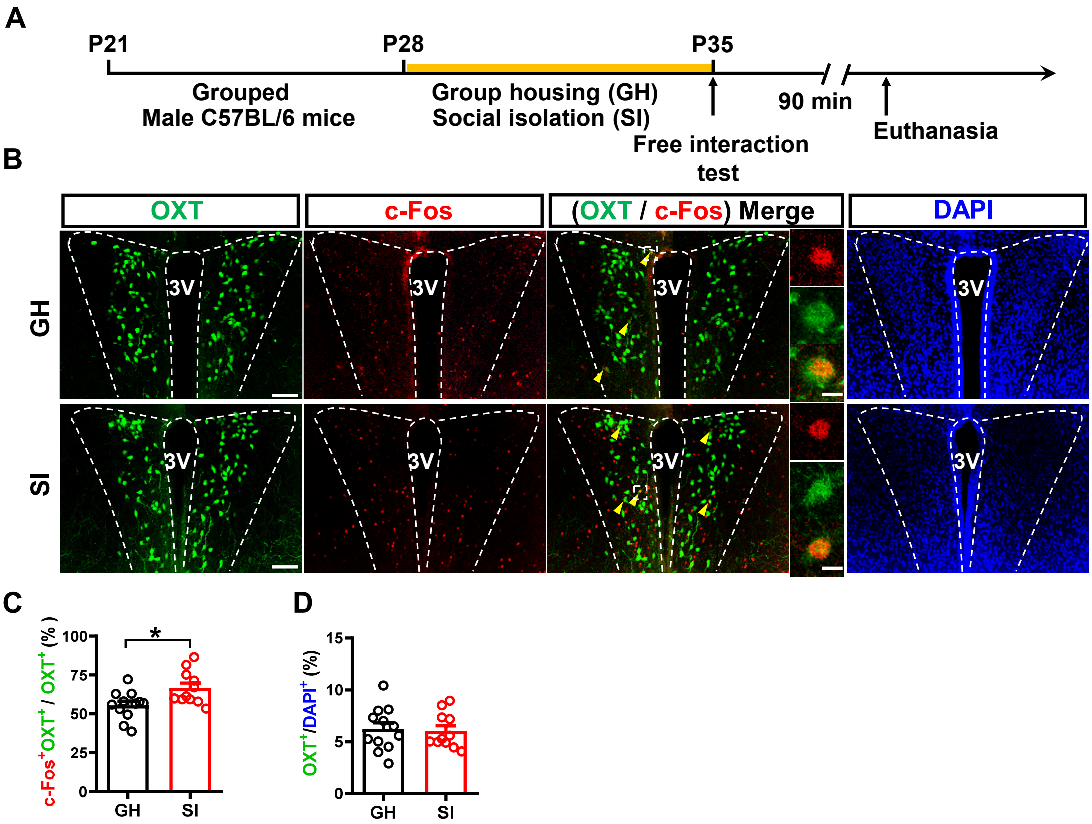

Supplementary Figure S2 Adolescent SI increases the activation of PVN oxytocin neurons in the free interaction test. **A** Schematic diagram illustrating the experimental pipeline. After weaning (P21), male mice were housed in groups of the same sex until P27 and housed either in groups or alone between P28 and P34. The free interaction test was performed on P35. Mice were euthanized 90 min after the test, and their brain was prepared for c-Fos expression analysis. **B** Representative images of c-Fos^{+} cells that are positive for oxytocin (OXT) in the PVN of male SI and GH mice. Immunofluorescent staining was performed in the tissues collected 90 min after the free interaction test. Scale bar, 200 μm. Yellow arrowheads indicate c-Fos^{+}OXT^{+} cells. c-Fos^{+}OXT^{+} cells were corroborated on higher magnification; scale bar, 10 μm. **C**, **D** Bar graphs with dots showing the percentages of c-Fos^{+}OXT^{+} / OXT^{+} [mouse number: GH: $n = 12$; SI: $n = 11$; two-tailed unpaired Student's t-test; $t_{(21)} = 2.68$, $P = 0.0139$, 95% CI (2.49 to 19.62); **C**] and OXT^{+} / DAPI^{+} [mouse number: GH: $n = 12$; SI: $n = 11$; two-tailed unpaired Student's t-test; $t_{(21)} = 0.24$, $P = 0.8115$, 95% CI (-1.84 to 1.46); **D**] in the PVN of SI and GH mice 90 min after the free interaction test. Data are presented as mean ± SEM. $^{*}P < 0.05$.
